# An effective solution to boost generation from waves: Benefits of a hybrid energy storage system integration to wave energy converter in grid-connected systems

**DOI:** 10.12688/openreseurope.14062.1

**Published:** 2022-03-23

**Authors:** Linda Barelli, Gianni Bidini, Dana Alexandra Ciupageanu, Andrea Ottaviano, Dario Pelosi, Federico Gallorini, Giacomo Alessandri, Mairead Atcheson Cruz

**Affiliations:** 1 Department of Engineering, University of Perugia, Via G. Duranti 1/A4, Perugia, 06125, Italy; 2 Power Engineering Faculty, University Politehnica of Bucharest, Splaiul Independentei 313, Bucharest, 060042, Romania; 3 VGA srl, Via Ugo Foscolo 1, Deruta, 06053, Italy; 4 Cruz Atcheson Consulting Engineers, Rua Fernando Namora 45 B 6 B, Lisboa, 1600 451, Portugal

**Keywords:** Wave Energy, WEC, Hybrid Energy Storage (HESS), Power Smoothing, PCC, grid-connected mode

## Abstract

**Background:** Wave energy represents one of the most promising renewable energies due to its great theoretical potential. Nevertheless, the electrical compliance of grid-connected systems is a great issue nowadays, due to the highly stochastic nature of wave energy.

**Methods:** In this paper, a Hybrid Energy Storage System (HESS) consisting of a Li-ion battery and a flywheel is coupled to a Wave Energy Converter (WEC) that operates in grid connected mode. The study is performed using real yearly wave power profiles relating to three different sites located along the European coasts. The Simultaneous Perturbation Stochastic Approximation (SPSA) principle is implemented as real-time power management strategy for HESS in wave energy conversion systems.

**Results:** Obtained results demonstrate how the proposed HESS and the implementation of the SPSA power management coupled to a WEC allow a reduction of more than 80% of power oscillations at the Point of Common Coupling (PCC), while proving the robustness of the developed management strategy over the investigated sites. Moreover, the average energy penalty due to the HESS integration results slightly higher than 5% and battery solicitation is reduced by more than 64% with respect to the flywheel solicitation, contributing to extend its lifetime.

**Conclusions:** HESS integration in renewable generation systems maximizes the WEC production while smoothing the power at the PCC. Specifically, flywheel-battery HESS together with the implemented power management strategy could provide a great flexibility in the view of increasing power production from waves, strongly mitigating the variability of this source while enhancing grid safety and stability.

## 1. Introduction

The growing world electricity demand and the threat of climate change due to greenhouse gases and pollution is currently pushing the research for new frontiers of energy production. In this context, renewable energy sources (RES) are widely exploited since they do not produce pollutant emissions and are largely available. Nevertheless, RES such as solar and wind energy show a stochastic nature that lead to an intermittent and fluctuating power production due to the variation of meteorological conditions. Therefore, RES are considered non-programmable power sources that can negatively affect grid stability and safety, system reliability, power quality and load management, as indicated in 1, 2. Considering the future increase in the installed capacity, the grid-connected RES will create a negative impact on the grid when fault or disturbance occurs. Consequently, the grid will have to manage higher rates of variable energy production while maintaining adequate voltage levels at the Point of Common Coupling (PCC). As the voltage is a local parameter, mitigating solutions should be designed and employed locally, enhancing not only the power quality indexes, but also providing support in fault conditions.

Since the 1970s, power production from sea and ocean waves has been studied, due to the major changes induced by the oil crisis of 1973 ^3^. Wind, as an indirect effect of the sun, blows across the surface of seas and oceans and generates waves, that can be exploited by Wave Energy Converters (WECs) to produce electricity ^4^. A review of WEC technologies developed over the years is presented in 5. Although WECs are currently considered immature technologies and none of them actually predominates over the others, the theoretical potential of the wave energy resource is very high (around 29,500 TWh/year) and can totally or almost cover global yearly energy consumption ^6, 7^. Maximum values can be predominantly registered between the 40° and 60° lines of latitude north and south, with a larger proportion in the southern hemisphere because of the large fetch lengths and relatively high winds, especially in the higher latitudes. One of the main issues affecting the commercial utilization of wave energy is related to the difficulty in integrating power from large WECs into the electricity grid because of the intrinsically high variability of the waves ^8^. In this context, different spectra have been developed to mathematically represent the wave elevation profile, using the principle of super-position of sinusoidal waves with different periods and heights. As analyzed in 4, a variety of idealized spectra have been suggested to represent a fully developed sea-state. Perhaps the Pierson-Moscowitz (PM) Spectrum ^9^ is the most widely used spectrum. It assumes that waves are in equilibrium with the wind when wind has been blowing across a sufficiently large expanse of water for a sufficiently long time. In this occurrence the sea state is fully developed and only wind speed affects the spectrum. Subsequently, the condition of not fully developed sea-state was performed by Hasselmann *et al*. in the Joint North Sea Wave Observation Project (JONSWAP), providing a refinement to the PM Spectrum based on the wind speed and fetch length ^10^. Further details on the above mentioned and other used wave spectra are reported in 11– 13.

Since the desired form of energy produced by the wave energy conversion process is electricity, the WEC systems have to encounter grid requirements and regulations. As described in 4, the main subsystems constituting a WEC are the following:

•     The prime mover, the system that absorbs wave power according to different principles as: point absorber, oscillating wave surge converter, submerged pressure differential, attenuator, rotating mass, oscillating water column and overtopping. It transfers forces and motions to both reaction and power take-off subsystems, described below, through suitable connections.

•     The Power Take-Off (PTO) subsystem, which converts the captured wave energy (by the prime mover) into electricity. The most common typologies are: hydraulic PTO, electro-mechanical PTO, linear generators, air turbine and low head water turbine.

•     The reaction subsystem, that maintains the WEC in position relative to the seabed (e.g. mooring system) and provides a reaction point for the PTO and/or support for the hydrodynamic subsystem(s) (e.g. fixed reference or support structure).

•     The control and monitoring subsystem, devoted to the WEC control and management. It mainly consists of the control software, power control system, sensors, devices for data transfer and the human interface.

As well as energy from other distributed generation sources, wave energy conversion systems produce power irregularly and intermittently and need to go through power conditioning before being fed into the electric grid as regulated in IEEE-1547-2003 ^14^. Three main requirements for a reliable WEC system have been suggested in 8, as it follows:

1. an efficient PTO system to convert mechanical power to electricity;
2. regularization of the unstable electricity to meet grid requirement or meet the electrical load if it is to be supplied to a standalone system;
3. the power electronics to ensure the quality of power at the user’s end.

In renewable energy generation, a promising solution to mitigate and reduce the fluctuating and intermittent behavior of RES consists in integrating energy storage systems (ESSs) in renewable power plants. ESSs provide the opportunity for the generation side to meet the level of power quality as well as the reliability required by the demand side, thanks to high flexibility, scalability and efficiency ^1, 15^. Moreover, ESSs can also provide emergency power and peak shaving functionality towards the grid. Therefore, ESS can actually provide an additional flexibility for RES penetration in the next years.

Among the several ESS technologies developed so far, battery energy storage systems (BESS) are usually employed for smoothing renewable power generation fluctuations ^10, 13^. However, many of the most widely used batteries, such as Li-ion batteries, are subjected to degradation due to electrochemical side reactions in anode, electrolyte and cathode ^16^. Moreover, the lifetime of many commercial BESS is strongly affected by harmful power spikes and depth of discharge (DoD), according to their specific cycle-to-failure (CTF) curves. Considering current BESS limits, researchers are investigating the use of short-term response ESSs, such as flywheel and super capacitors, aiming to absorb/provide instantaneous power spikes for wind power smoothing. Nevertheless, neither flywheels nor supercapacitors are able to provide adequate storage capacity for long term periods, not exceeding seconds or few minutes.

The main features of flywheel energy storage system (FESS), are: fast responsiveness, high efficiency, long cycling life and high power densities ^17^. In comparison to other high power ESSs such as supercapacitors (SCs), FESSs are cheaper and have a higher lifespan, although low specific energies and standing losses are non-negligible aspects. Indeed, supercapacitors are often investigated in similar applications for fast dynamic power regulation (coupled to batteries in hybrid systems), as shown in 18. As current limitations, SCs have very low specific energies (up to 5–10 Wh/kg, lower than FESSs) and high daily self-discharge rates. Furthermore, SC technology has very high capital costs (0.01–0.015$/F, predicted for a large production) ^19^. This implies that FESSs and SCs are usually used for power modulation (i.e. short-term energy storage).

Consequently, hybridization among different technologies can bring significant achievements, since Hybrid Energy Storage Systems (HESS), including multiple storage devices complementary to each other, are able to cope with storage requirements for different timeframes, merging the positive features of base-technologies and extending their application ranges ^20^.

In the scientific literature, many research activities have been focused on the integration of ESSs in wave energy farms, coupled to different kind of WECs. Hazra and Bhattacharya ^21^ propose a hybrid energy storage system comprising of a battery and ultra-capacitor for power smoothing of oscillating wave energy. In 21 it is demonstrated how HESS minimizes the grid side converter rating improving grid stability, while claiming the cost-effectiveness of this solution in comparison to a battery and ultra-capacitor not-hybrid solution. In 22 a new control strategy for power smoothing was applied to a wave farm coupled to a FESS. The authors obtained grid losses reduction by 51%, improving the energy efficiency of the power network. According to 23, the integration of FESSs into a WEC plant achieves a reduction of 50% in power oscillations, covering 85% of the frequency excursions at the grid, on the basis of real power generation profiles delivered to the electric grid. Another noteworthy research employed FESS in order to enhance dynamic stability of an integrated offshore wind and marine-current farm ^24^. In 25 an interesting study concerning FESS for wave power leveling was carried out. Among the several ESS technologies for power smoothing, supercapacitors were also analyzed ^26, 27^, due to the similar features with respect to FESSs, as mentioned before. Fang *et al*. ^28^ presented a coordinated and stable control for a hybrid energy storage system constituting a battery and a flywheel with the purpose to inhibit power fluctuations when wave generator works in grid connected mode.

In the present paper, a hybrid energy storage system including a Li-ion battery and a flywheel has been developed and implemented with the purpose of reducing power oscillations produced by WEC system and sent to the grid. A specific and optimized power management, based on the Simultaneous Perturbation Stochastic Approximation (SPSA) algorithm ^29– 33^, has been implemented aiming to minimize power fluctuations sent to the grid. The study presented in 28 is focused on the assessment of voltage and frequency stability in grid connected mode, while no quantitative information is provided for what concerns power oscillations reduction at the PCC.

Therefore, the main objective of the present work is to demonstrate how HESS features can improve power quality at the PCC while minimizing the related energy penalty due to the storage integration. Dynamic simulations were carried out in MATLAB® Simulink (R2019a) environment considering three different wave power profiles. Xcos is suggested as an alternative open source software that can be used to replicate the simulation method. All the details concerning Simulink to Xcos conversion in order to replicate the implemented methodology are available here.

In Section 2, the wave energy fundamentals and the methodology regarding data selection and power profile generation are described. A statistical characterization of the generated annual power profiles for the three sites has been realized both considering their yearly features and daily ones as reported in Section 3. A description of HESS modelling and sizing is illustrated in Section 4– Section 5. Section 6 reports the simulation results achieved over several daily profiles extracted as the most representative of the wave yearly production. Furthermore, the advantages of the SPSA power management strategy are highlighted resulting in about 80% power fluctuations reduction at the PCC with respect to the wave-generated profile, with an average energy penalty of only 5.3%.

## 2. Methods

In this section, the applied methodology for the considered case studies is discussed.

### 2.1 Wave energy fundamentals

Being a consequence of wind, wave nature is inherently stochastic and the conversion of this energy is extremely complex due to the hydrodynamic processes present in the diffraction and radiation of the waves as they propagate to shore ^34^. The power per unit width of a wave front is given by 34 ( Equation 1):

$$P=\frac{1}{64}\frac{\rho g^{2}}{\pi }H_{s}^{2}T_{e}\;(1)$$

Where:

*P* is the power per unit width (kW/m);

*ρ* is the density of sea water (kg/m ^3^);

*g* is the acceleration due to gravity (m/s ^2^);

*H _s_* represents the significant wave height (m);

*T _e_* is the wave energy period (s).

*T _e_* value differs from the wave peak period *T _p_* in accordance with the considered spectrum used for hindcasts. For instance, *T _e_* = 0.857 *T _p_* considering the PM spectrum ^35^. 

### 2.2 Generation of the power profiles

Wave energy data were obtained from thirty-year wave hindcast data validated through experimental measurements ^36^. The hindcast was performed using the National Oceanic and Atmospheric Administration WAVEWATCH III model data (see here for more details), and was driven by winds from the NCEP Climate Forecast System Reanalysis (CFSR), a coupled reanalysis of the atmospheric, oceanic, sea-ice and land data (access to the CFSR data requires registration via the website). 

**Figure 1.**
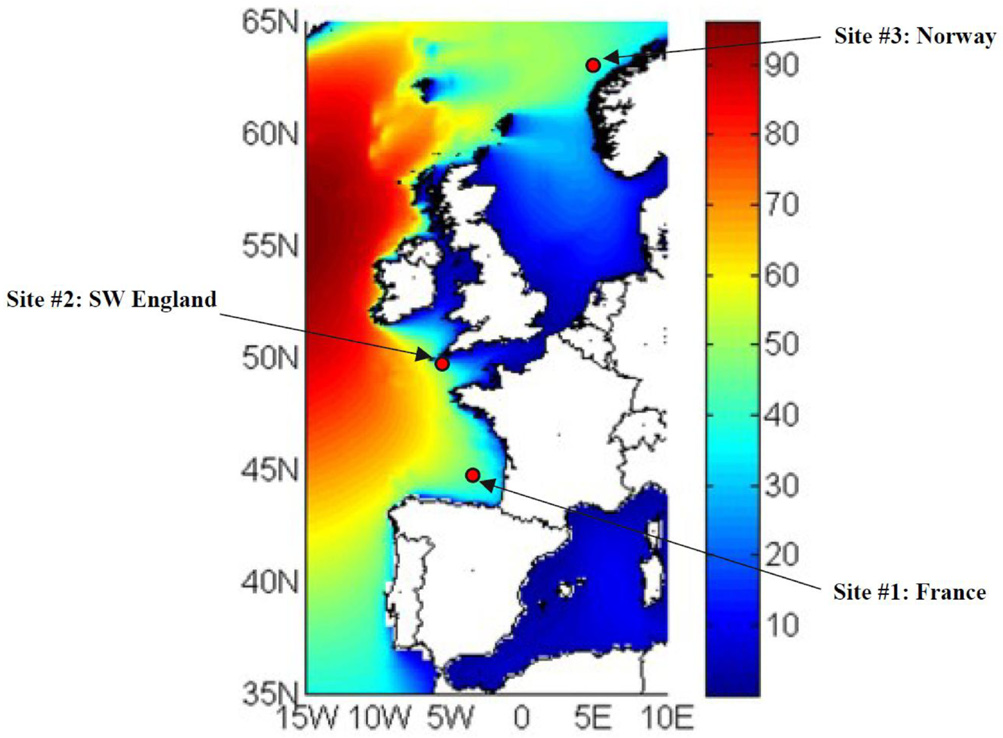
Selected locations for simulating the wave power in input to the hybrid energy storage system.

Three different sites located in the European region are considered ( Figure 1), with an annual mean wave power that ranges between 30 and 50kW/m. The selection of these sites was made in the framework of the IMAGINE project in order to match the available wave power at the WEC inputs with the power of the PTO system developed during the project.

The measured wave occurrences related to the three sites were elaborated in the form of yearly scatter matrices by aggregating the samples with same wave height *H _s_* and period *T _p_*. The number of sea states resulting for each location is:

•   Site 1, located in France: 143 sea states;

•   Site 2, located in England: 144 sea states;

•   Site 3, located in Norway: 132 sea states.

**Figure 2.**
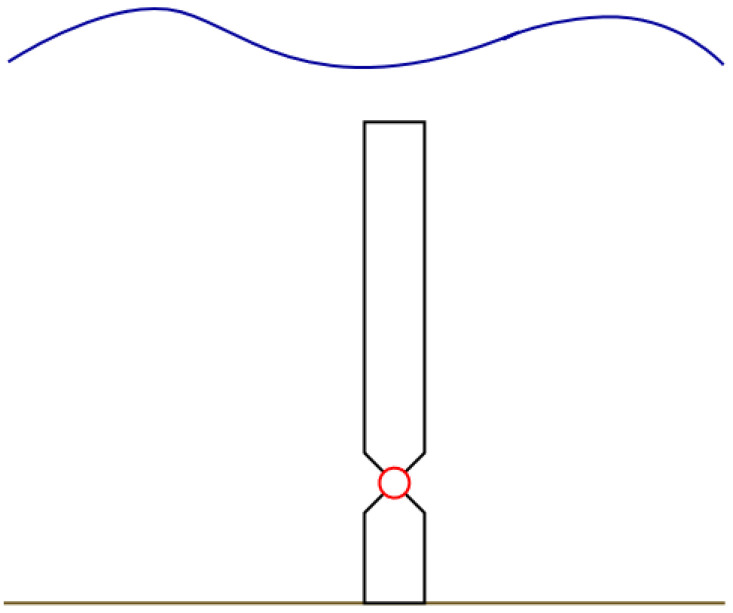
The Oscillating Wave Surge Converter (OWSC) wave energy converter type, bottom mounted, submerged.

Figure 2 represents the considered Oscillating Wave Surge Converter, OWSC, WEC type with two PTOs having a rated power of 250 kW each, for the calculation of the generated electrical power.

For each cell of the scatter matrix (with a specific spectrum, wave period *T _p_* and wave height *H _s_*) a numerical simulation was performed by using the WEC-Sim (Wave Energy Converter SIMulator) software ^37^, an open-source WEC simulation tool developed in MATLAB/Simulink environment.

With the purpose to build a dataset to study the dynamic response of the HESS in reducing power oscillations at the PCC, a proper sample time step of 0.1 s was chosen. This provides the main features needed to characterize the power take off (PTO) according to the specific input conditions. Specifically, the following parameters related to the PTO input axis were evaluated from the simulation tool:

•   Acceleration;

•   Speed;

•   Stroke;

•   Load.

On the basis of these data, it is possible to determine the instantaneous power in output from the PTO system, through the following equation ( Equation 2):

P=ηvD(2)

Where:

*η* represents PTO system (from input axis to DC bus) efficiency; 

*υ* is the instantaneous speed value at the PTO expressed in m/s;

*D* is the instantaneous force at the PTO input axis, expressed in N.

In this work it has been assumed a PTO efficiency equal to 80%, in line with data obtained by the same system in similar applications ^38^.

Three random vectors of the instantaneous electric power were generated in MATLAB® for each site, in order to evaluate any quantitative difference due to the randomization process. Each vector represents the instantaneous power profile generated in a year, with a time step of 0.1 s. Such vectors were constructed by randomly concatenating each 30 minutes power data sequence obtained through simulation, globally repeating each sequence for a number of times equal to the related occurrences number.

### 2.3 Random vectors comparison

**Figure 3.**
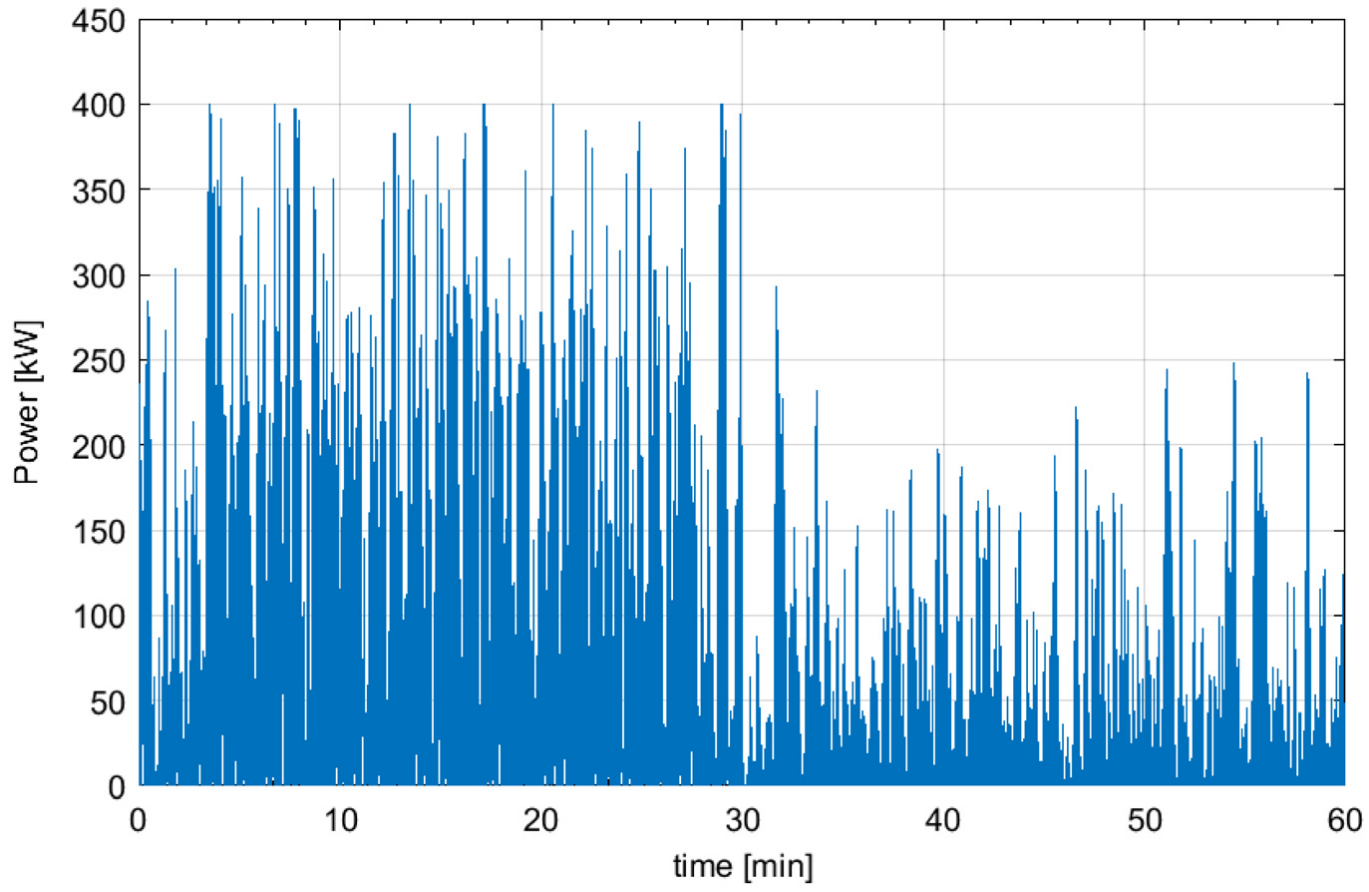
Wave generated power profile for 1 hour.

Based on the wave characteristics depicted in Section 2.2, three random yearly power vectors are generated for each site, having a 0.1 s time step. Figure 3 depicts as example a part (1 hour) of one of the generated electric power profiles. It must be emphasized how the power trend is very oscillating and fluctuating.

The differences among the three vectors are analyzed for each site, considering as a principal comparison criterion the distribution of the instantaneous power ramp $R_{wave}^{t}$ , expressed by Equation (3) as the difference between the power values $P_{wave}^{t}$ and $P_{wave}^{t-1}$ at two consecutive time steps, with a 1 s window. This quantity is considered as an objective of further research, since it is strictly related to the smoothing of the power output generated by the wave energy converter.

$$R_{wave}^{t}=P_{wave}^{t}-P_{wave}^{t-1}\;(3)$$

In reference to the first site, it is remarked that the instantaneous power ramp is always under 3.8 kW/s, regardless of the random vector considered. This threshold reduces to 3.4 kW/s for the second site, while the maximum value of the instantaneous power ramp for the third site grows up to 4.2 kW/s.

Table 1 presents a comparison in reference to the power ramp 90% Cumulative Density Function (CDF) threshold value, averaged for the three vectors and for each site. It is emphasized that the maximum deviation from the average (if comparing, for each site, a single random vector to the average value) doesn’t exceed 0.5%. Thus, no significant difference arises among the three random vectors. So, further analyses are made based on the first vector generated for each site.

**Table 1.** 90% CDF threshold values comparison.

Site	Average power ramp [kW/s]	Percentage power ramp deviation (relatively to the average) [%]
1	3.235	0.36
2	2.675	0.41
3	3.451	0.26

## 3. Statistical characterization of wave energy generation

### 3.1 Yearly features

In the following section, a brief comparison among the power generation sites is presented in terms of the variability features for few characteristic quantities. The assessments are carried out on the random yearly power vectors selected (one for each site) at the previous stage, highlighting the diversity of the generation spectrum at each site and pointing out their suitability for a comprehensive analysis on wave energy integration in interconnected systems. Therefore, it is emphasized that the representative quantities are considered based on their relevance in properly describing the operating conditions on yearly basis. Specifically, the selected quantities to describe the behavior of the wave generation at each site are:

- generated energy;
- daily mean power;
- the power generation daily bandwidth, calculated as the difference between the daily maximum and minimum recorded values;
- the ratio between the daily bandwidth and the mean power, being a measure of how wide the spread of the power trend around the mean is;
- the instantaneous power ramp, determined as the difference between two consecutive values, introducing the highest perturbations to the grid (in terms of fluctuating power profile at the interconnection point), being at the same time the most stressful condition for the energy storage devices.

**Figure 4.**
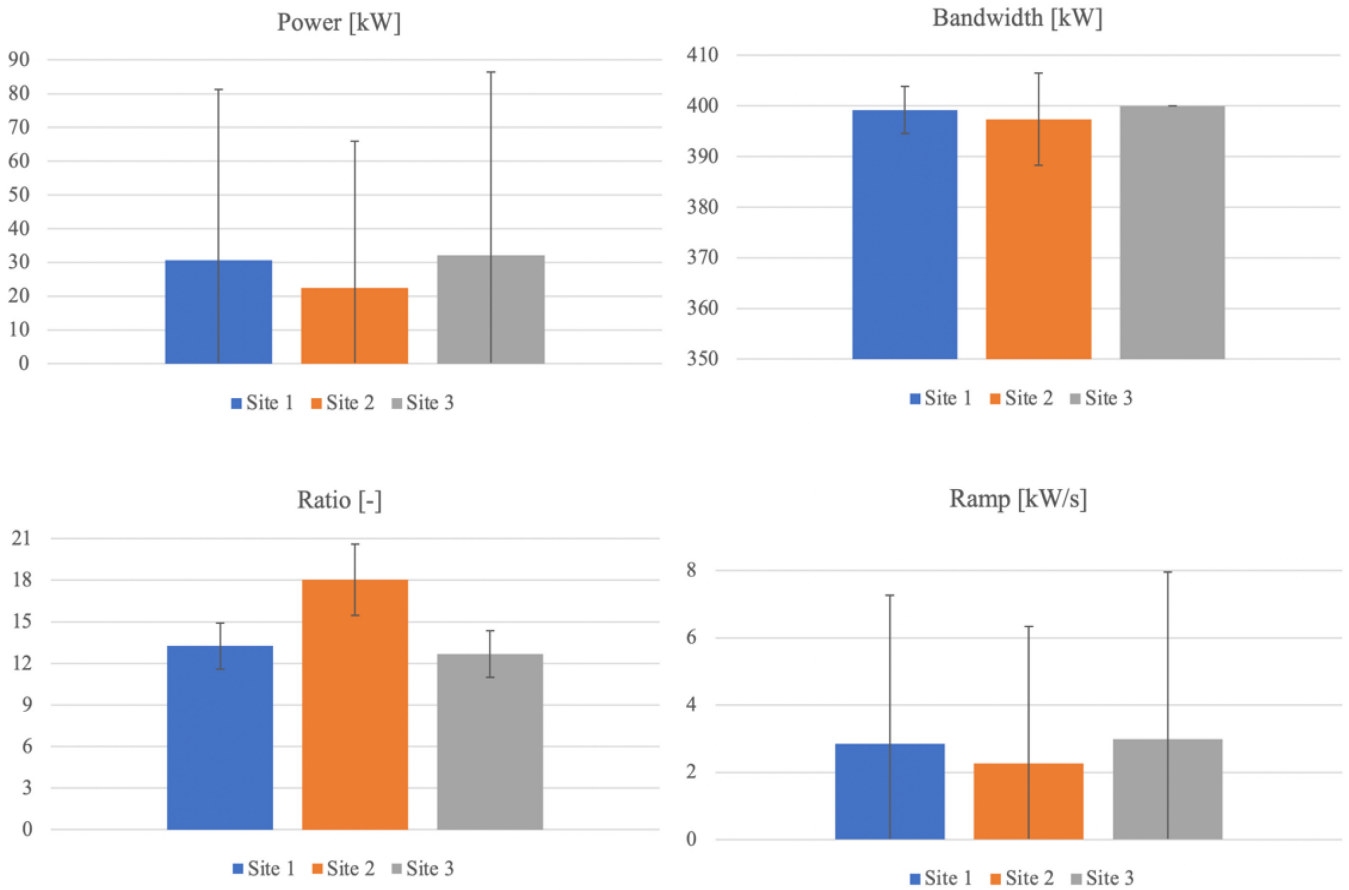
Statistics comparison.

Figure 4 depicts a comparison among the three sites for all the quantities introduced above, a detailed discussion in reference to each one being provided below.

***a. Site 1 – France.*** The generated energy over one year in the first site reaches 256.85 MWh, resulting the second among the three sites. According to Figure 4 (blue columns), it is noted that both power and ramp distribution results are very widespread, and that the deviation from the mean exceeds more than 65%. For what concerns the bandwidth, the deviation is reduced, representing around 1% of the mean, highlighting that in most of the days, the difference between maximum and minimum power is approximately the same and equal to the maximum possible. As a consequence of quite different distributions of the quantities involved, the deviation corresponding to the ratio between the bandwidth and mean power gets up to 12.6% relatively to the mean.

***b. Site 2 – England.*** The yearly generated energy adds up to 188.97 MWh for the second site, being the smallest amount among the three. Both power and ramp distribution spread are very wide also for this site as evident in Figure 4 (orange columns), outranking the first one. Specifically, the deviation nearly doubles the mean for both quantities. The bandwidth deviation reaches around 2.3% of the mean, being doubled if compared to the first site. The deviation corresponding to the ratio between the bandwidth and mean power reaches 14.2% relatively to the mean, being the highest among the three sites. Therefore, it can be concluded that the second site shows the highest variability and the lowest production (with up to 30% smaller than the others).

***c. Site 3 – Norway.*** The annual energy generation at the site located in Norway reaches 269.12 MWh, the highest value among the three sites. It is noted that both power and ramp distribution is very widespread, according to Figure 4 (grey columns), close to the values evaluated for the first site, the deviation exceeding the mean by up to 69% (65% for site 1). The bandwidth shows a particular behavior in this site, being remarkable that every day the power generation varies between 0 and the maximum possible power (400 kW). So, the bandwidth is constant and maximal. The deviation corresponding to the ratio between the bandwidth and mean power gets up to 13.3% relatively to the mean.

### 3.2 Daily features

Subsequent to the characterization of the yearly profiles randomly generated with a time step of 0.1 s, it is intended to select some representative days in order to run numerical simulations for sizing a hybrid energy storage system for each considered installation site. The target of integrating this system coupled to the wave energy plant is to smooth the power profile exported to the grid. In order to obtain simulation profiles compatible with the response time of the energy storage devices, control time step and ease the computational burden, the yearly profiles are averaged over 1 s time frame. Consequently, simulation profiles (with 1 s time step for 24 hours) are extracted from the yearly profile and employed in simulation. The representative days are selected based on the statistics previously defined, as it follows:

- Day 1: maximum bandwidth;
- Day 2: maximum mean power;
- Day 3: maximum bandwidth to mean power ratio;
- Day 4: minimum bandwidth to mean power ratio;
- Day 5: maximum mean ramp.

**Figure 5.**
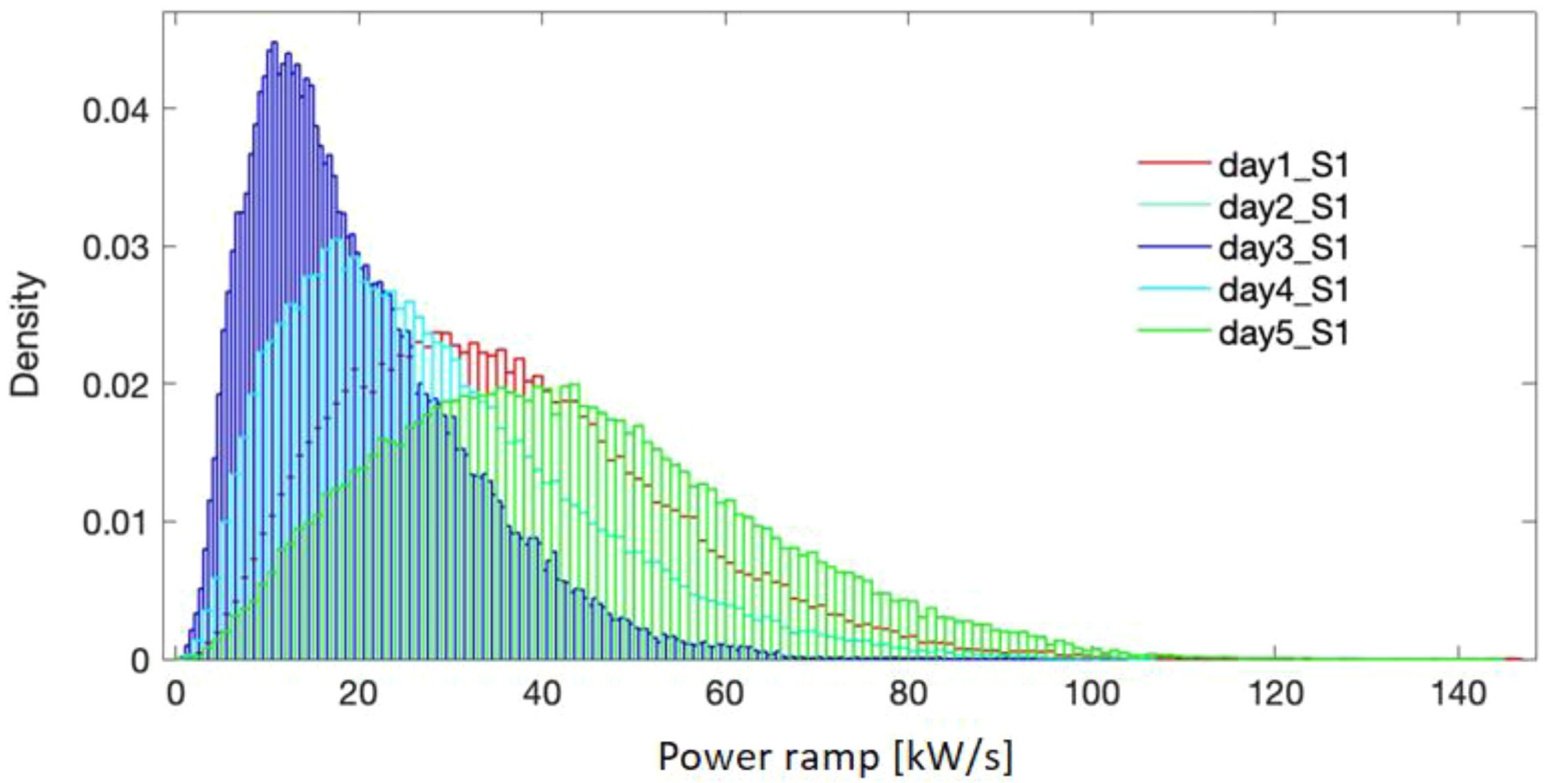
Probability density for the selected days - site 1.

Figure 5– Figure 7 present the probability distribution for the selected representative days in all the three sites. It is evidenced that they cover the domain of power variation with different densities, making therefore the simulation results significant in the sizing process. Numerical characteristic values corresponding to each site are listed in Table 2, Table 3 and Table 4.

**Figure 6.**
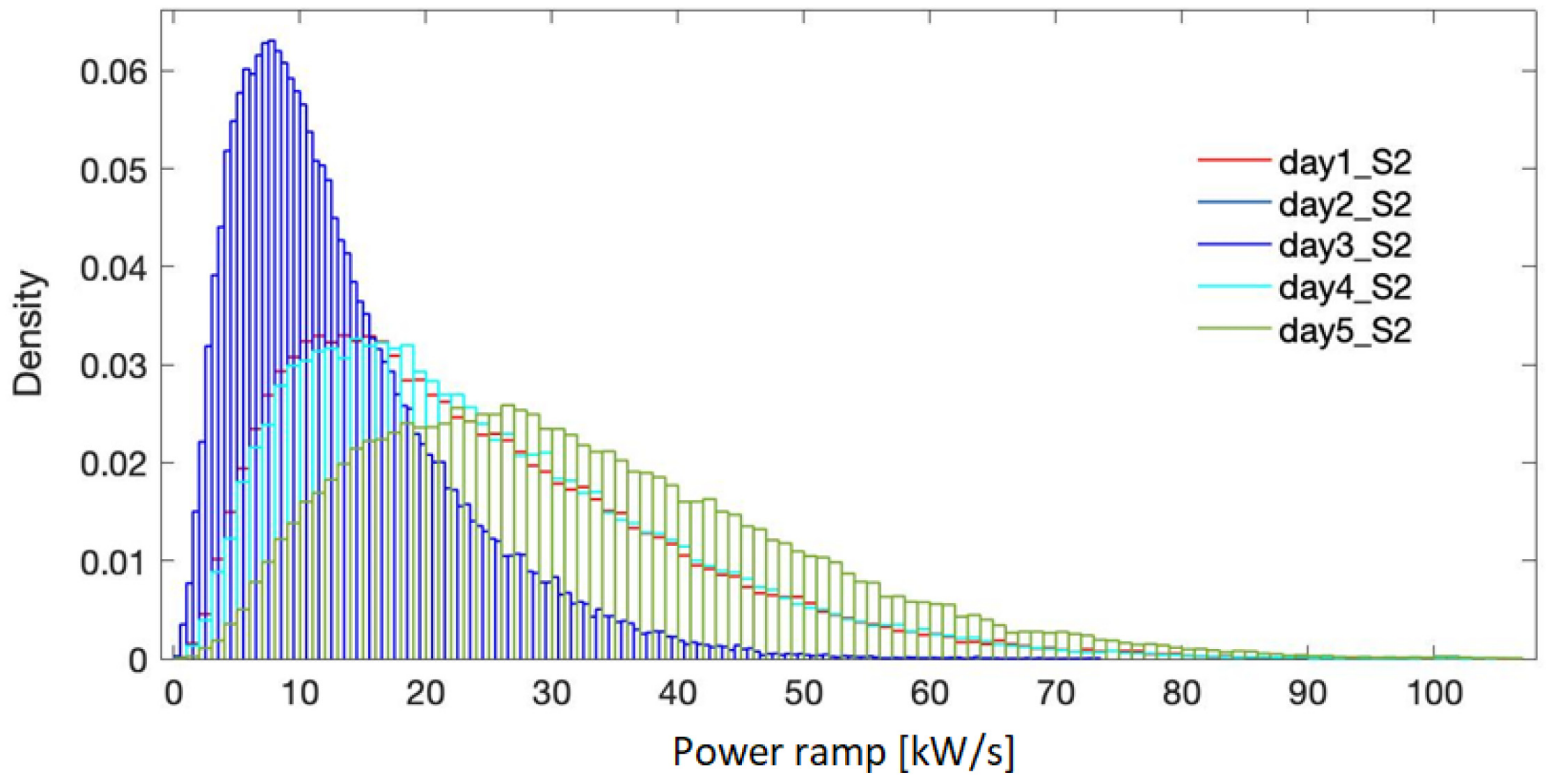
Probability density for the selected days - site 2.

**Figure 7.**
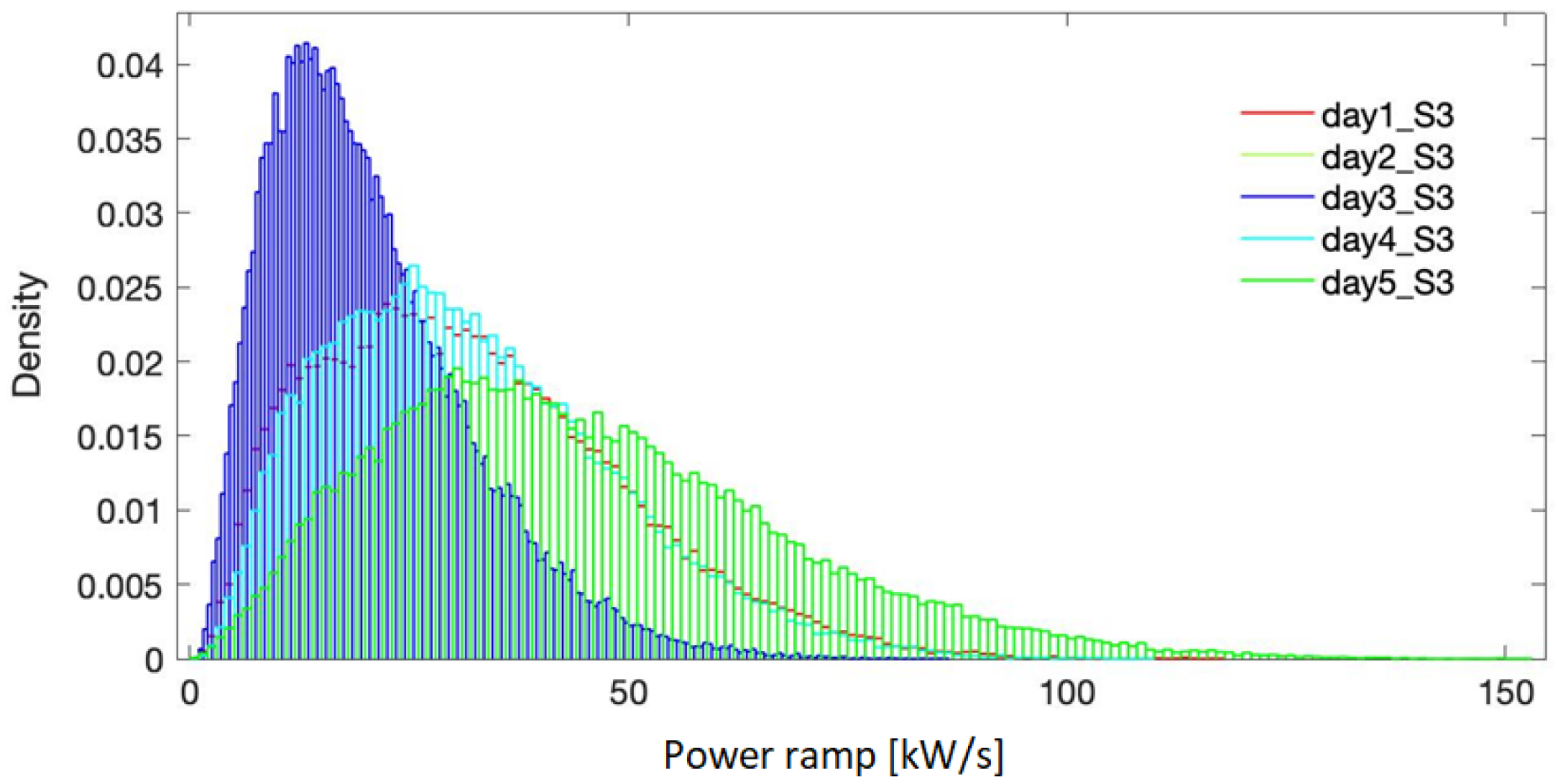
Probability density for the selected days - site 3.

**Table 2.** Characteristics of the selected days – site 1.

*Day*	*Daily average* *power [kW]*	*Energy* *generated [kWh]*	*Maximum &* *Minimum power [kW]*	*Bandwidth* *[kW]*	*Bandwidth/* *Average power*	*Daily Average* *power ramp [kW/s]*
1	36.01	864.15	146.19 / 1.16	145.03	4.03	1.59
2	42.97	1 031.3	144.71 / 0.47	144.24	3.36	1.73
3	19.94	478.58	101.88 / 0.60	101.29	5.08	0.89
4	28.17	676.08	106.18 / 0.83	105.35	3.74	1.18
5	42.97	1 031.3	144.71 / 0.47	144.24	3.36	1.73

**Table 3.** Characteristics of the selected days – site 2.

*Day*	*Daily average* *power [kW]*	*Energy* *generated [kWh]*	*Maximum &* *Minimum power [kW]*	*Bandwidth* *[kW]*	*Bandwidth/* *Average power*	*Daily Average* *power ramp [kW/s]*
1	24.09	578.17	105.30 / 0.74	104.56	4.34	1.34
2	32.08	770.01	106.39 / 0.67	105.72	3.29	1.46
3	13.31	319.39	73.38 / 0.39	72.99	5.48	0.73
4	24.47	587.39	105.65 / 0.62	105.03	4.29	1.11
5	32.08	770.01	106.39 / 0.67	105.72	3.29	1.46

**Table 4.** Characteristics of the selected days – site 3.

*Day*	*Daily average* *power [kW]*	*Energy* *generated [kWh]*	*Maximum &* *Minimum power [kW]*	*Bandwidth* *[kW]*	*Bandwidth/* *Average power*	*Daily Average* *power ramp [kW/s]*
1	31.94	766.48	117.69 / 1.02	116.66	3.65	1.46
2	44.36	1 064.7	152.90 / 0.31	152.59	3.44	2.09
3	20.49	491.81	86.82 / 0.45	85.92	4.18	0.91
4	31.91	765.82	109.54 / 0.34	109.20	3.42	1.45
5	44.36	1 064.7	152.90 / 0.31	152.59	3.44	2.09

**Figure 8.**
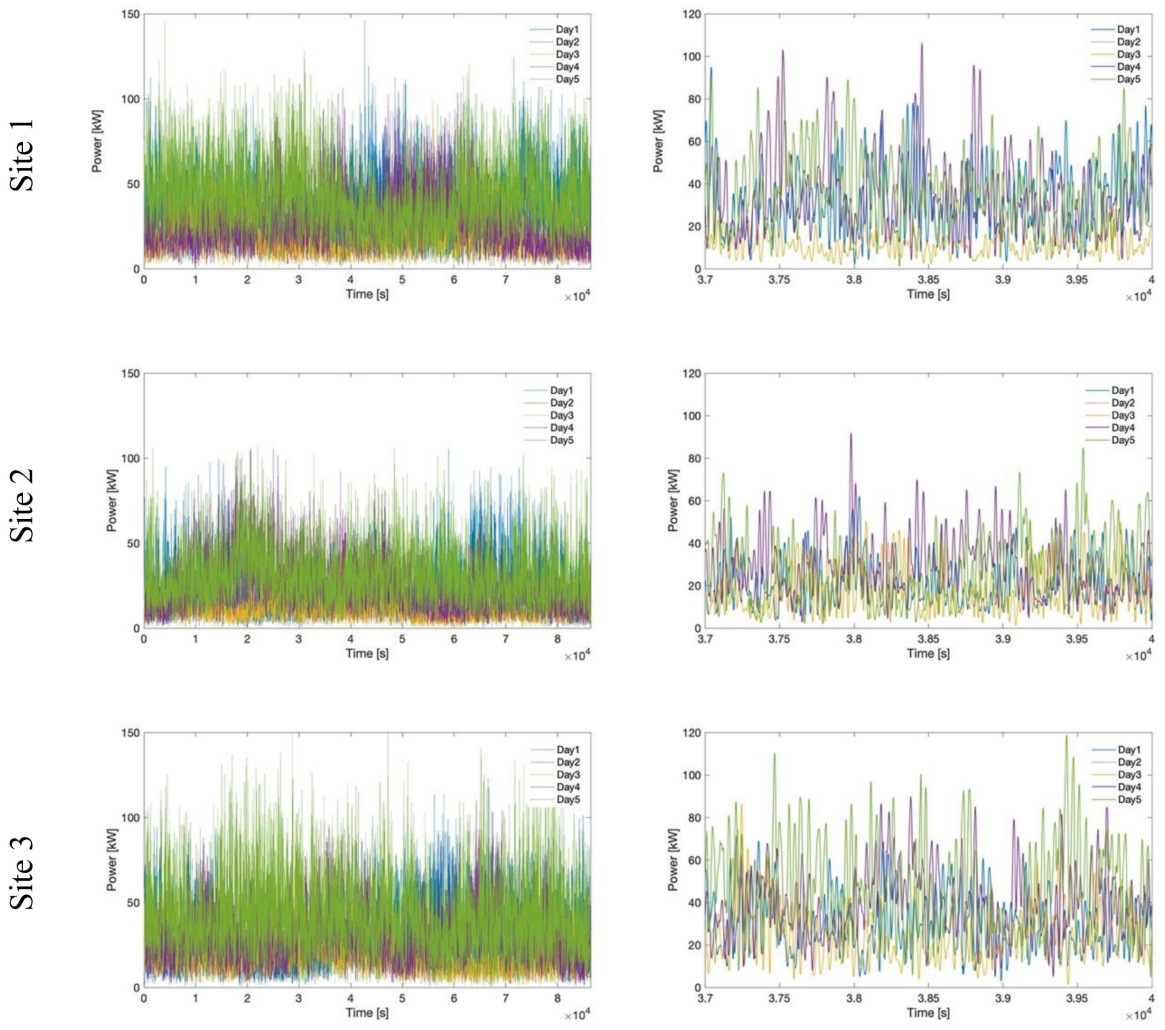
Comparison of power profiles for all selected days and sites.

The power profiles selected for simulation for each site are represented in Figure 8, together with a detail on the same time window. The provided representation allows us to highlight the differences in amplitude and shape among the representative days of each location and, moreover, the three sites, while allowing to consider a wide range of profiles, with high/low generation, strong/weak fluctuation.

## 4. Hybrid energy storage system modeling and control

### 4.1 Modeling considerations

**Figure 9.**
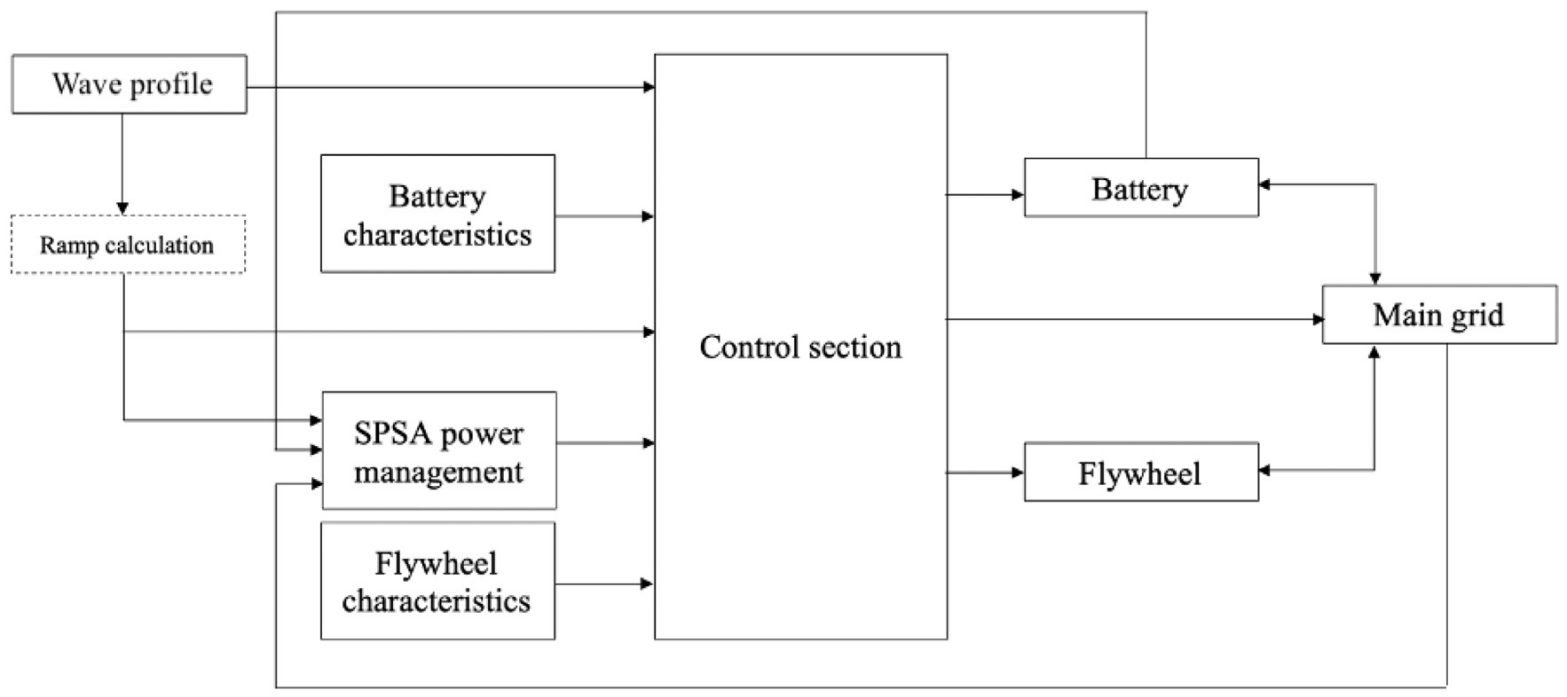
Simulink model outline.

Figure 9 depicts the model of the hybrid energy storage system (HESS) as developed in the Matlab/Simulink environment. The power profile delivered by the wave converter enters the SPSA power management module together with the power processed by the battery and exchanged with the grid at the previous time step. This to perform the real time management of both flywheel and battery aiming to the management targets indicated in the next section 4.2. The outcomes provided by the SPSA power management module are processed by the main control section to take into account constraints due to the technical features of both battery and flywheel and current operating conditions related to their state of charge. Thus the instantaneous values of the three shares, in which the power produced by the WEC is shared by the SPSA algorithm among the battery, the flywheel and the grid, are imposed.

The implemented LiFePO _4 _battery pack is characterized by a maximum charge current of 1C A and maximum discharge current of 3C A, with a nominal voltage of 420 V. The low-speed flywheel is a mechanical one, equipped with low-friction mechanical bearing and housing under vacuum. The rotor is cylindrical and made of steel, while its rotational speed varies from 366 to 890 rad/s. The torque vs. speed saturation curve and the efficiency curve for the driving electrical machine are based on values measured on a real machine and then properly scaled.

### 4.2 Power management strategy

For the on-line management optimization the SPSA algorithm is chosen, among several stochastic ones, to overcome drawbacks of conventional algorithms ^35, 36^ since it has proven to be an effective stochastic optimization method in a wide range of practical applications ^33^. The SPSA algorithm exhibits fast convergence for the global optimization problem of an unknown functional form of systems performance, i.e. the loss function, with a reduced computational burden ^31^. Moreover, its implementation doesn’t need of a mathematical uncertainty model.

**Figure 10.**
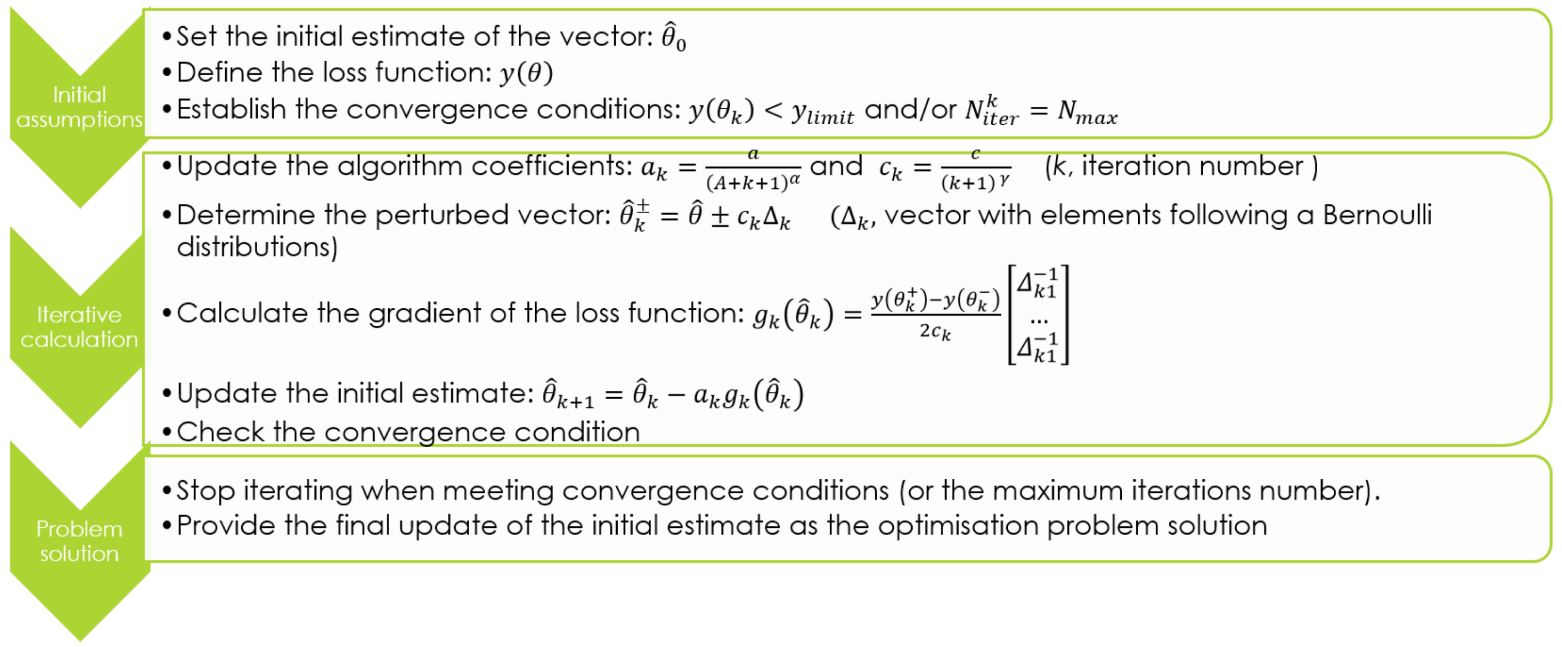
Simultaneous Perturbation Stochastic Approximation (SPSA) algorithm description.

Figure 10 describes the SPSA algorithm iterative procedure for the optimal solution determination. It moves from an initial estimation of the parameters vector ( $\hat{\theta }$ ) and the definition of the loss function ( *y*( *θ*)).

The optimal solution is searched iteratively. At each iteration the algorithm coefficients *a _k_*, *c _k_* are updated ( *A, a, c, α, γ* values are set as visible in Table 5 to ensure calculation convergence according to literature ^38, 39^) and all the parameters of vector $\hat{\theta }$ are simultaneously perturbed. Thus two estimates ( ${\hat{\theta }}_{k}^{\pm }$ ) of the parameters vector are determined. These estimates are used to evaluate the gradient of the loss function, *g _k_* ( ${\hat{\theta }}_{k}$ ). The updated estimation ( ${\hat{\theta }}_{k+1}$ ) of the parameters vector is assessed in consideration of *g _k_* ( ${\hat{\theta }}_{k}$ ) and a _k_. The loss function is evaluated in reference to ${\hat{\theta }}_{k+1}$ and the convergence condition is checked.

**Table 5.** Algorithm parameters.

Parameter	Value
A	10
A	2.1127e ^–5^
C	1e ^–5^
α ^30, 41^	0.602
γ ^30, 41^	0.101
Maximum iterations	100

The iterative calculation is stopped when the convergence conditions (or the maximum iterations number) is achieved, and the final update of the initial estimate is provided as the optimisation problem solution. This corresponds to a vector of parameters which ideally brings the gradient of the loss function, *g _k_* ( ${\hat{\theta }}_{k}$ ), to zero or to a convergence threshold.

Similarly to the power management strategy proposed by the authors in 40, the problem formulation here presented implements through Equation (4)– Equation (8) the SPSA algorithm. This implementation aims to the real-time calculation of the optimal shares of power exchanged with the flywheel, the battery and delivered to the grid in managing the oscillating wave power profile. Specifically, the SPSA algorithm manages the difference between the current value of the wave power and the previous value of the power injected at the PCC (Δ *P* as expressed in Equation (4)) sharing it among the battery (q _batt_), the flywheel (q _fw_) and the grid (q _grid_). Consequently the instantaneous exchanged power values can be determined according to Equation (5), permanently taking into account maximum battery and flywheel capacity and power. To ease the smoothing process, the difference Δ *P* is pre-processed using a moving average filter with a one-hour window length.

$$\text{Δ}P=P_{wave}^{t}-P_{grid}^{t-1}\;(4)$$

$$\begin{array}{c} P_{batt}^{t}=q_{batt}\cdot \text{Δ}P\\ P_{fw}^{t}=q_{fw}\cdot \Delta P\\ P_{grid}^{t}=q_{grid}\cdot \text{Δ}P \end{array}\;(5)$$

The target is the smoothing of both power profiles sent to the grid and managed by the battery (extending its lifetime ^20, 42^). This is achieved thanks to flywheel ramping capability and fast response, exploited to compensate the fast variations of produced power, while smoothing the profiles imposed to the battery. The corresponding mathematical formulation implements a multi-object loss function of the SPSA algorithm, as indicated in Equation (6) in terms of the weighted sum of two objectives.

$$y^{k}(\theta )=w_{1}\cdot y_{1}^{k}(\theta )+w_{2}\cdot y_{2}^{k}(\theta )\;(6)$$

Where:

1. The smoothness of the power profile exchanged with the grid is modeled through Equation (7) as the ratio between the power delivered to the grid at the current (t) and previous (t-1) timestep.
$$y_{1}^{k}(\theta )={\left(\frac{q_{grid}\cdot \text{Δ}P}{P_{grid}^{t-1}}\right)}^{2}\;(7)$$
2. Similarly, Equation (8) expresses the smoothness of the battery power profile.
$$y_{2}^{k}(\theta )={\left(\frac{q_{batt}\cdot \text{Δ}P}{P_{batt}^{t-1}}\right)}^{2}\;(8)$$
3. *w*_1_ = *w*_2_ = 0.5, that is the same relevance is considered for the two objectives.

Starting from an initial estimate of vector θ, the q shares are iteratively determined, following the SPSA principle illustrated in Figure 10. Specifically, q _batt_ and q _grid_ are calculated according to the SPSA algorithm, while q _fw_ results imposing the power balance. The convergence condition is imposed accordingly with the loss function expressed by Equation (6). Furthermore, 100 is fixed as the maximum number of iterations to avoid any loop in the convergence procedure.

## 5. Hybrid energy storage system sizing

**Figure 11.**
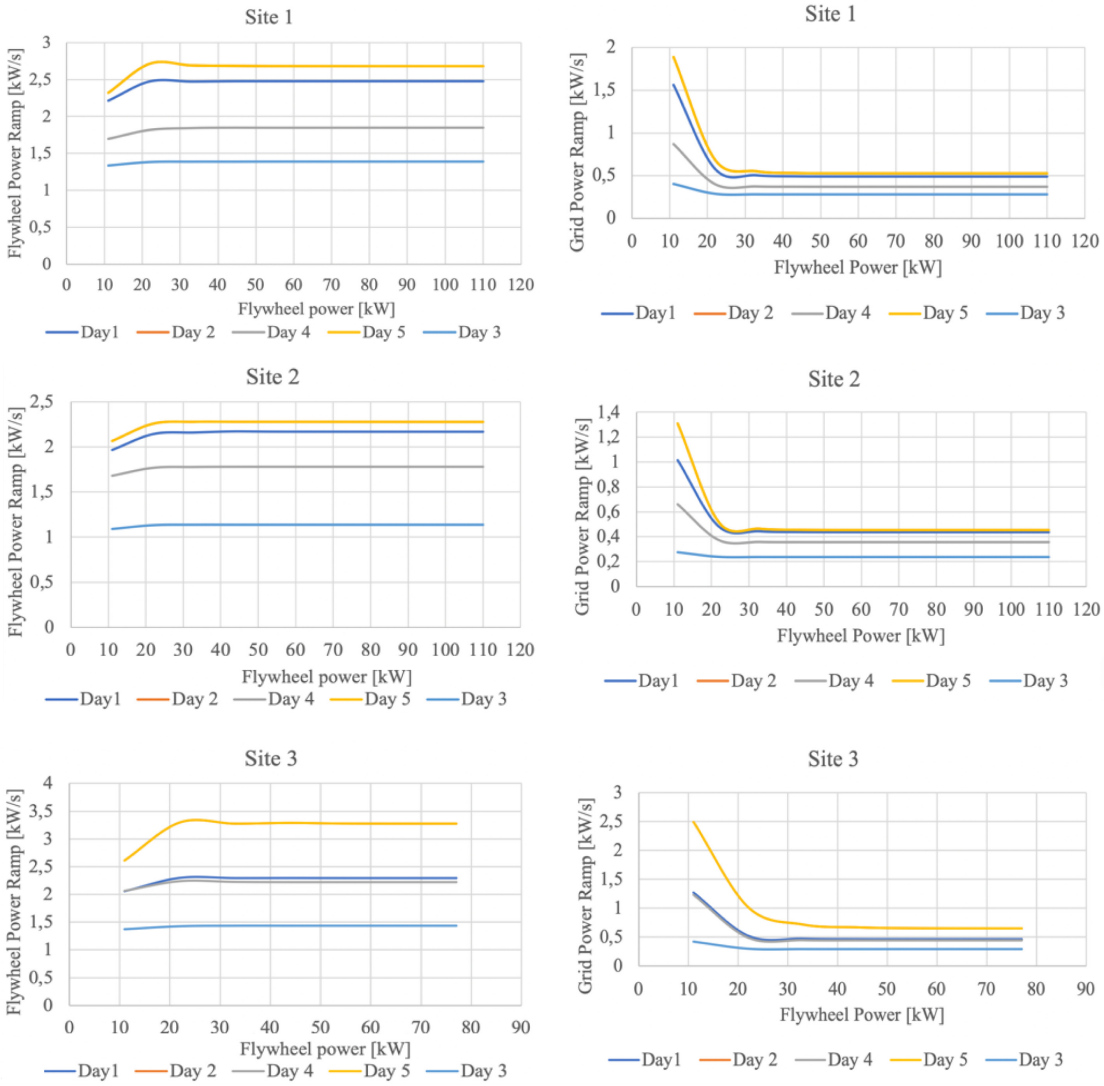
Sensitivity analysis in reference to flywheel sizing; all trends correspond the 90% threshold value of the CDF evaluated for the power ramp.

The sizing procedure mainly follows two stages:

- a first assumption in reference to both storage devices is made starting from the cumulative distribution of the power ramp calculated according to Equation (3). It is noted that the storage devices are customized one at a time, aiming to avoid/minimize saturation in power and capacity; so, the flywheel rating (both in power and capacity) is adjusted first, assuming a very large battery, since the flywheel is more adequate to fast response operation (i.e. power smoothing). The capacity and maximum power of the flywheel are determined so that the saturation objective is achieved. Further, the capacity of the battery is adjusted (so, considering the C-rate also the power), following the same objective in terms of saturation.
- The second stage, resulting in the final sizing of the HESS components (in particular the flywheel) implies a sensitivity analysis performed relatively to the power ramp mitigation capability, varying the flywheel maximum power. As it emerges from Figure 11, there is a point on each graph where the flywheel is able to ensure a maximum power ramp mitigation towards the grid. It is remarked that the trends plotted in Figure 11 correspond the 90% threshold value of the CDF evaluated for the power ramp. It can also be observed as for values lower than 20 kW flywheel power, the power ramp at the PCC is too high and by increasing the flywheel power it can still be mitigated. Furthermore, for flywheel power values larger than 33 kW, the power ramp reduction at the PCC is insignificant, the power ramp conveyed to the flywheel being approximately constant for all the three investigated sites. The resulting final sizing of the HESS is presented in Table 6. It is noteworthy that the differences in terms of battery capacity are correlated with the total amount of energy generated over one year, as discussed in Section 3.1. Site 2 and Site 3 exhibit, in fact, the lowest and the largest annual energy production respectively.

**Table 6.** Hybrid energy storage system (HESS) components final sizing.

	Site 1		Site 2		Site 3	
	Power [kw]	Capacity [kWh]	Power [kw]	Capacity [kWh]	Power [kw]	Capacity [kWh]
Flywheel	33	2.1	33	2.1	33	2.1
Battery	66	22	60	20	72	24

## 6. Discussion

Following the sizing process, the power smoothing performance towards the grid, related to coupling the HESS to the wave energy plant, is assessed for all three sites. The power management strategy implemented is based on the simultaneous perturbation stochastic approximation principle, as detailed in 40, 43.

**Figure 12.**
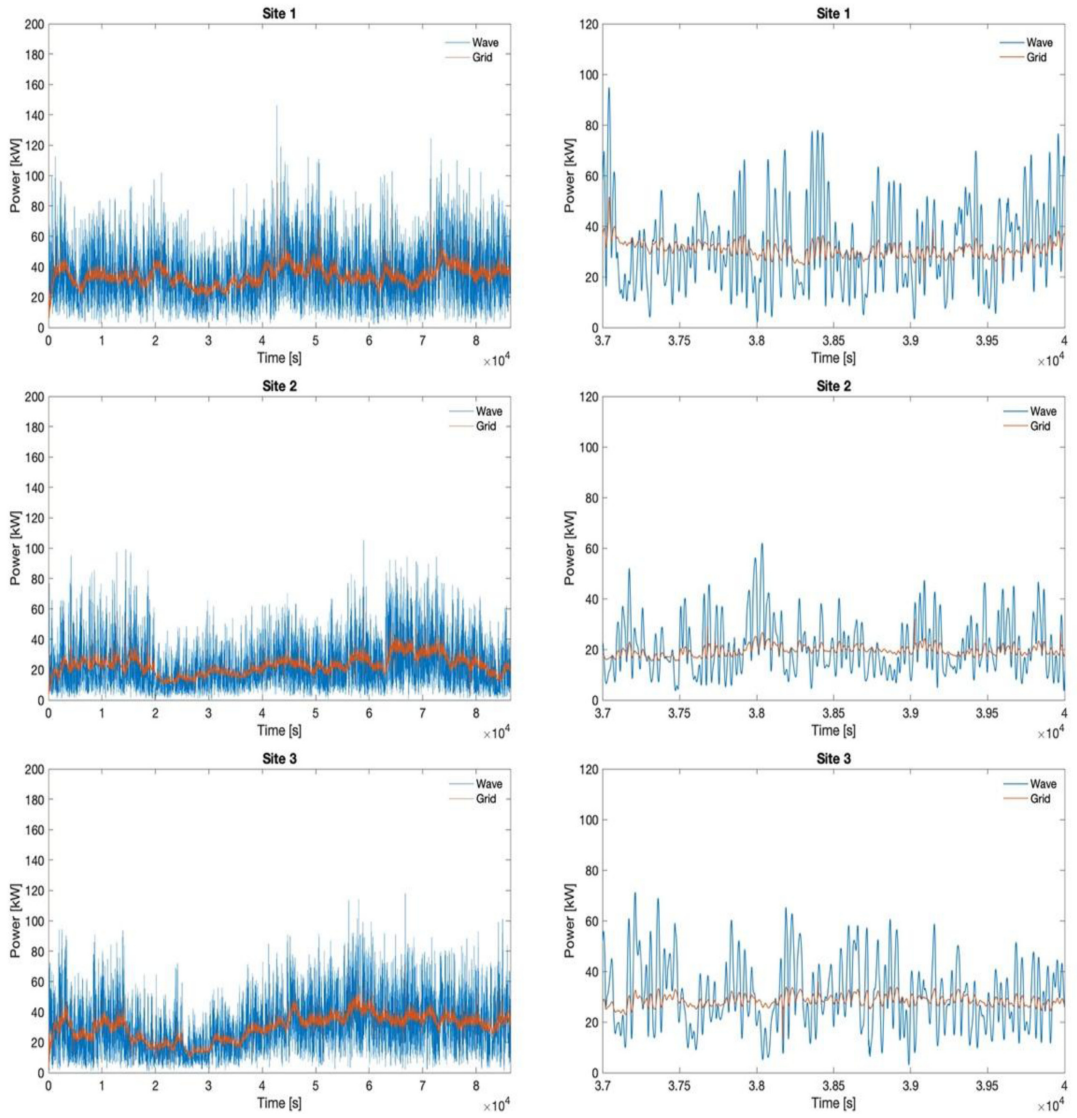
Original wave profile and smoothed grid profile for all sites in day 1.

Figure 12 depicts the power profile at the PCC compared to the original one generated by the wave converters. The right side shows a detailed comparison between the wave and the grid power in a random interval from Day 1 (characterized by the maximum bandwidth). It is remarked that the fluctuations are substantially reduced.

**Figure 13.**
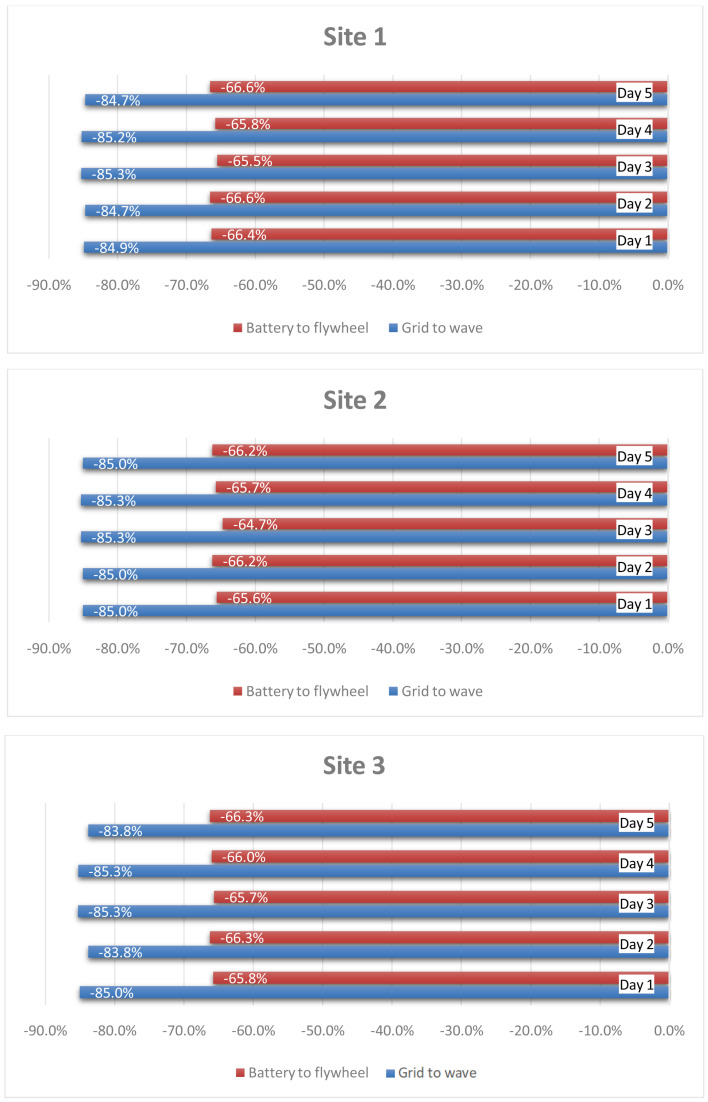
Power ramp reduction.

Figure 13 quantifies the smoothing of the fluctuation achieved towards the grid thanks to the HESS coupling with wave energy converters and the implemented stochastic power management algorithm. The evaluation is made in reference to the 90% CDF threshold. Moreover, a comparison is conducted also between the battery and flywheel, to demonstrate the reduction in fluctuation of the battery input power profile with respect to the flywheel one. The latter is obtained thanks to different management of the two devices in terms of operating modes, implemented according also to their technical features and restrictions. It can be noticed that the results are uniform, the HESS (with the implemented power management algorithm) achieving approximately the same performances in all three sites under all considered operating conditions.

The energy exchanges are evaluated below under the simulated conditions for all three sites. Based on the results of absorbed and released energy by the battery and flywheel, their operational efficiencies are determined. An average battery efficiency of 97% is found over all conducted simulations, while a 79.5% efficiency is evaluated for the flywheel. For what concerns the ratio between the wave energy produced and the amount processed by the HESS, it is emphasized that it varies within a range of 5% among the three sites, specifically between 78% and 83%. Regarding the global energy losses, the total energy delivered to the grid amounts, as the average value among the three sites, to 94.7% of the wave converters production, thus with an average energy penalty of 5.3%. These values are consistent with the operation energy efficiency determined for the battery and the flywheel, considering that only 17–22% (taking into all the three sites) of the produced energy is managed by the HESS being not directly delivered to the grid. 

## 7. Conclusions

Among the RES, wave energy arouses a broad interest in research because of its very high theoretical potential. Even if only few of the WEC technologies have reached commercial maturity, many efforts are addressed to increase the efficiency of generation, one of the greatest issues is related to the grid connection. Therefore, many research activities deal with ESS integration with renewables in order to improve power quality and grid safety. In this work, flywheel-Li-ion battery HESS is employed for power smoothing towards the grid, aiming to maximize power generation and, at the same time, reducing harmful fluctuations for grid stability. The advantages of SPSA power management on HESS allow a reduction of more than 80% of power oscillations at the PCC, with an average energy penalty slightly higher than 5%. Furthermore, simulations demonstrate that flywheel features can extend Li-ion battery lifespan, decreasing battery solicitations of more than 64% with respect to flywheel fluctuations.

## Data availability

### Underlying data

The National Oceanic and Atmospheric Administration WAVEWATCH III model data used in this study is available here: https://polar.ncep.noaa.gov/waves/ensemble/download.shtml

The Climate Forecast System Reanalysis (CFSR) dataset used in this study is openly available upon registration here: https://rda.ucar.edu/datasets/ds093.1/#!access

Zenodo: An effective solution to boost generation from waves: benefits of HESS integration to wave energy converter in grid-connected systems. https://doi.org/10.5281/zenodo.5993310 ^44^.

This project contains the following underlying data:

- Flywheel_power_ramp_site_1.csv (flywheel power saturation data for site 1)
- Flywheel_power_ramp_site_2.csv (flywheel power saturation data for site 2)
- Flywheel_power_ramp_site_3.csv (flywheel power saturation data for site 3)
- Grid_power_ramp_site_1.csv (grid power saturation data for site 1)
- Grid_power_ramp_site_2.csv (grid power saturation data for site 2);
- Grid_power_ramp_site_3.csv (grid power saturation data for site 3);
- Statistics_power.csv (data relating to Figure 4 for the three considered sites);
- Statistics_bandwidth.csv (bandwidth data relating to Figure 4 for the three considered sites);
- Statistics_ratio.csv (ratio data relating to Figure 4 for the three considered sites).
- Statistics_ramp.csv (power ramp data relating to Figure 4 for the three considered sites).#

### Extended data

Zenodo: An effective solution to boost generation from waves: benefits of HESS integration to wave energy converter in grid-connected systems. https://doi.org/10.5281/zenodo.5993310 ^44^.

This project contains the following extended data:

- wave_sim.mat (in this file the main simulations data are saved and can be opened through the free software GNU Octave, loading it in the workspace with the command: load ‘wave_sim.mat’. The profiles of Figure 8 can be reproduced in GNU Octave loading wave_sim.mat and plotting the following files: day1_S1, day2_S1, day3_S1, day4_S1, day5_S1, day1_S2, day2_S2, day3_S2, day4_S2, day5_S2, day1_S3, day2_S3, day3_S3, day4_S3, day5_S3).
- 1hour_profile.mat (in this file the hourly power profile of site 1 is saved and can be opened through GNU/Octave loading it in the workspace with the command: load ‘1hour_profile’).
- pdf_site1_data.mat (in this file the data related to the probability density function of site 1 are saved and can be opened through GNU/Octave loading it in the workspace with the command: load ‘pdf_site1_data’).
- pdf_site2_data.mat (in this file the data related to the probability density function of site 2 are saved and can be opened through GNU/Octave loading it in the workspace with the command: load ‘pdf_site2_data’).
- pdf_site3_data.mat (in this file the data related to the probability density function of site 3 are saved and can be opened through GNU/Octave loading it in the workspace with the command: load ‘pdf_site3_data’).
- Smoothing_site_1.mat (in this file the data of the smoothed power and generated power relating to site 1 are saved and can be opened through GNU/Octave loading it in the workspace with the command: load ‘Smoothing_site_1.mat’).
- Smoothing_site_2.mat (in this file the data of the smoothed power and generated power relating to site 2 are saved and can be opened through GNU/Octave loading it in the workspace with the command: load ‘Smoothing_site_2.mat’).
- Smoothing_site_3.mat (in this file the data of the smoothed power and generated power relating to site 3 are saved and can be opened through GNU/Octave loading it in the workspace with the command: load ‘Smoothing_site_3.mat’).

Data are available under the terms of the Creative Commons Attribution 4.0 International license (CC-BY 4.0).
