# Supplementary material for: An effective solution to boost generation from waves: Benefits of a hybrid energy storage system integration to wave energy converter in grid-connected systems
Source: Open Res Eur. 2023 Sep 4;2:40. Originally published 2022 Mar 23. [Version 2] doi: 10.12688/openreseurope.14062.2 (PMC11109555; doi:10.12688/openreseurope.14062.2)
Supplement: Peer review history

# Reviewer report: Reviewer response for version 2

**DOI:** 10.21956/openreseurope.17795.r39296

**Published:** 2024-05-10

**Authors:** Demián García-Violini

**Affiliations:** 1 Universidad Nacional de Quilmes, Bernal, Argentina

The paper introduces a novel strategy aimed at enhancing power quality within wave energy converters through the integration of hybrid energy storage systems. While the simulation results exhibit promising outcomes, a critical evaluation reveals several areas warranting enhancement, notably in terms of methodological depth, comprehensive cost analysis, and rigorous energy sizing evaluation. Overall, the manuscript presents some insights, yet it requires substantial refinement to meet the rigorous standards expected. Strengthening technical rigour and addressing identified shortcomings are imperative steps toward elevating the manuscript's scholarly contribution. In general, the study fails to provide sufficient details for replication, hindering the reproducibility of the results. A better presentation of dynamical models involved, for simulation and assessment, related, for example, to the flywheel and DC bus, must be included. Find below some crucial comments and concerns:

 - To the best of this reviewer's knowledge, the use of supercapacitors is more common in the literature than flywheels. A cost analysis, a concrete and specific analysis to support the decision of considering flywheels, i.e. a mechanical-based energy storage system, must be provided. 

 - It is worth noting that the reference provided for the literature review on WEC systems (Reference 5) is outdated (2010), overlooking the significant advancements that have occurred in WEC technology over the past 15 years. Updating this reference to include more recent studies would ensure a more comprehensive review of the current state-of-the-art in WEC systems: Please update this reference with a more recent study.

 - The power synthesis in Fig. 3 is confusing. Have waves been synthesised for each of the points (Fig 1) using the PW spectrum? Then, based on the synthesised waves, are power profiles, such as the one shown in Fig. 3, estimated? If so, what variables of peak period, significant height, etc., are considered? This aspect is presented very loosely, and needs to be reinforced. Just in case, and given the subsequent sections in the manuscript, if this procedure is related to the effect introduced by the flywheel, this is not realistic at all. 

 - The following paragraph (before Eq. (3)) "The differences among the three vectors are analysed for each site, considering as a principal comparison criterion the distribution...". It is not clear how you plan to smooth and 'ramp' the power profile. In essence, what's the plan for physically dissipating the power profile? What's the benefit or requirement for this procedure? The expression Eq. (3) is a filtered version  of the power profile, how is this definition made? This aspect must be clarified.

 - There is a misuse of the term bandwidth (Fig 4), which must be related to spectral bandwidth (Hz or rad/s), however the manuscript considers this terms as a power measure. Correct and clarify this crucial aspect.

 - There is no model presented for the Li-io battery nor the flywheel. This aspects make the study impossible to replicate or assess. 

 - No model is presented for the Li-ion battery or the flywheel. This aspect makes the study impossible to replicate or assess. This has to be discussed and explained in the manuscript. At a minimum, there should be a discussion to support the decision to omit the inclusion of a mathematical model. Similarly, the DC bus is not explained, including its efficiency factor. 

 - What are the columns in Figs. 8 and 12? Please, and in general, Figures must be clearly explained, presented, and discussed.

 - Can the authors provide more detailed information on the efficiency of the HESS connected power converter and how it impacts the overall performance of the system?

 - How was the cost and energy density comparison between different storage systems, specifically Li-ion batteries and supercapacitors, conducted, and what were the key findings?

Is the study design appropriate and does the work have academic merit?

Partly

Is the work clearly and accurately presented and does it cite the current literature?

Partly

If applicable, is the statistical analysis and its interpretation appropriate?

Partly

Are all the source data underlying the results available to ensure full reproducibility?

No

Are the conclusions drawn adequately supported by the results?

Partly

Are sufficient details of methods and analysis provided to allow replication by others?

No

Reviewer Expertise:

Control systems; dynamical systems; Wave Energy Systems.

I confirm that I have read this submission and believe that I have an appropriate level of expertise to confirm that it is of an acceptable scientific standard, however I have significant reservations, as outlined above.

# Reviewer report: Reviewer response for version 2

**DOI:** 10.21956/openreseurope.17795.r34906

**Published:** 2023-10-26

**Authors:** Luigi Piegari, Marzio Barresi

**Affiliations:** 1 Politecnico di Milano, Milan, Italy

Several comments of this reviewer have been neglected (or rejected).

 Even if the paper is improved, the reviewers remain of their original idea that some parts could be better explained (e.g., the SoC diagrams could have been added to the results, the proposed sizing procedure is not optimal, i.e. a smaller FESS could give the same advantages, ...).

Is the study design appropriate and does the work have academic merit?

Partly

Is the work clearly and accurately presented and does it cite the current literature?

Yes

If applicable, is the statistical analysis and its interpretation appropriate?

I cannot comment. A qualified statistician is required.

Are all the source data underlying the results available to ensure full reproducibility?

Partly

Are the conclusions drawn adequately supported by the results?

Partly

Are sufficient details of methods and analysis provided to allow replication by others?

No

Reviewer Expertise:

Power electronics for renewables, batteries and automotive, electrical drives, electrochemical storage modeling

We confirm that we have read this submission and believe that we have an appropriate level of expertise to confirm that it is of an acceptable scientific standard, however we have significant reservations, as outlined above.

# Response

**Published:** 2023-11-07

**Authors:** Federico Gallorini

**Affiliations:** VGA srl, Italy

Below further details are provided according to the specific comments of the reviewers, relative to the need of integration of SoC diagrams obtained in simulation and details relative to the flywheel sizing with particular reference to its maximum power. SoC profiles, obtained through simulation in reference to the day 1 (similarly to what depicted in Figure 12) for all the investigated sites, can be integrated in the Results section. Figure 13 can be added to the manuscript.

 Figure 13 [a], [b], [c]. Instantaneous battery SoC and flywheel speed for: a) site 1, b) site 2 and c) site 3 in day 1.

 With regards to flywheel sizing, it is highlighted as it doesn't follow an optimization procedure, being implemented based on the methodology described in Section 5 and illustrated in Figure 11. Specifically, a sensitivity analysis on power ramp at the PCC varying flywheel maximum power is performed for all the investigated 5 days for all the sites. As evident from Figure 11, grid power ramp (in terms of 90% CDF assessed on the basis of the simulated power ramp profiles relative to the investigated 5 days for each site) reduces increasing flywheel maximum power (starting from 11 kW) and it stabilizes at around 33 kW for all the considered days in the three sites. A further increase over such a power does not contribute to a further reduction in the grid power ramp; this indicates that higher flywheel powers are unnecessary for the studied application.

# Reviewer report: Reviewer response for version 1

**DOI:** 10.21956/openreseurope.15154.r29630

**Published:** 2022-07-28

**Authors:** Arvind Parwal, Andrej Savin

**Affiliations:** 1 Department of Engineeing Science, Uppsala University, Uppsala, Sweden

**Arvind Parwal**

**Alstom AB, Stockholm, Sweden**

**Member, WECANet COST Action 17105**

**Andrej Savin**

**Uppsala University, Uppsala, Sweden**

 The paper is an interesting example of how a hybrid energy storage system including a Li-ion battery and a flywheel has been developed and implemented with the purpose of reducing power oscillations produced by WEC system and sent to the grid.

 Dynamic simulations were carried out in MATLAB® Simulink environment considering three different wave power profiles.

 Obtained results demonstrate how the proposed HESS and the implementation of the SPSA power management coupled to a WEC allow a reduction of more than 80% of power oscillations at the Point of Common Coupling (PCC).

**Comments are the following:**

- Author presents only one drawing for only one WEC system in Fig.2. It may be interesting to include in the section "Introduction" several concepts for wave energy conversion and the principle of energy conversion that can use a Hybrid Energy Storage System (HESS).
- The efficiency of HESS connected power converter is missing. This data shall be included for maintaining the readership of the Journal.
- Cost and Energy density of FESS shall be presented, as shown on pg. 4 for SCs.
- The sizing of energy storage is not clear, this data shall be added that could show the purpose of the study is fully covered using FESS/HESS.
- Power converters control modelling and used topologies are also missing in the study. It would be beneficial for the readers to have this information available.
- Li-ion batteries are being complemented with Flywheel energy storage, this study lacks the information about the SOCs of the batteries and the power losses during the operation.
- Efficiency of the DC-DC power converter could be presented in buck and boost mode for the three sites investigated.
- Authors write “In this paper, HESS consisting of a Li-ion battery and a flywheel is coupled to a Wave Energy Converter (WEC) that operates in grid connected mode”.

It is interesting what the model of flywheel-Li-ion battery HESS looks like. Constriction and connection principal to WEC.

- Authors write on Page 3 “The Power Take-Off (PTO) subsystem, which converts
  the captured wave energy (by the prime mover) into electricity. The most common typologies are: hydraulic PTO, electro-mechanical PTO, linear generators, air turbine and low head water turbine”.

Technical aspects: for example how to connect flywheels to linear generators? From my point of view, it is almost impossible to do. But even if it succeeds, it will be a complicated mechanical system that takes resources and becomes costly.

- It is good to adjust the scale in Fig.12 so that empty spaces are not so large.

- The authors have laid out in a very reasonable manner how simulations demonstrate that flywheel features can extend Li-ion battery lifespan, decreasing battery solicitations of more than 64% with respect to flywheel fluctuations.

- The article referenced some relevant references on HESS. However few more references shall be included showing the HESS studies (Li-on Batteries/SCs/Flywheel/Fuel Cells).

- An overall overview of the Electrical power system used in this study shall be included.

The paper presents an interesting approach to reducing power fluctuations. It is a good platform for evaluating offshore experiments.

 Data is provided in Matlab. However, reproducing the experiment in more detail would significantly improve the paper. The paper is relevant for indexing.

**We confirm that we have read this submission and acknowledge that it is of an acceptable scientific standard.**

Is the study design appropriate and does the work have academic merit?

Yes

Is the work clearly and accurately presented and does it cite the current literature?

Yes

If applicable, is the statistical analysis and its interpretation appropriate?

I cannot comment. A qualified statistician is required.

Are all the source data underlying the results available to ensure full reproducibility?

Partly

Are the conclusions drawn adequately supported by the results?

Yes

Are sufficient details of methods and analysis provided to allow replication by others?

No

Reviewer Expertise:

Wave power systems for renewable energy conversion; HESS and Grid Integration; Power quality, Flickers and transients analysis at the PCC; linear and rotational wave energy converters; electromechanical design

We confirm that we have read this submission and believe that we have an appropriate level of expertise to confirm that it is of an acceptable scientific standard.

# Response

**Published:** 2023-08-01

**Authors:** Linda Barelli

**Affiliations:** Department of Engineering, Universita degli Studi di Perugia, Perugia, Umbria, Italy

POINT TO POINT ANSWERS TO REVIEWERS The paper is an interesting example of how a hybrid energy storage system including a Li-ion battery and a flywheel has been developed and implemented with the purpose of reducing power oscillations produced by WEC system and sent to the grid. Dynamic simulations were carried out in MATLAB® Simulink environment considering three different wave power profiles. Obtained results demonstrate how the proposed HESS and the implementation of the SPSA power management coupled to a WEC allow a reduction of more than 80% of power oscillations at the Point of Common Coupling (PCC).   Comments are the following:

- Author presents only one drawing for only one WEC system in Fig.2. It may be interesting to include in the section "Introduction" several concepts for wave energy conversion and the principle of energy conversion that can use a Hybrid Energy Storage System (HESS).

We considered this suggestion in the review process of the manuscript

-  The efficiency of HESS connected power converter is missing. This data shall be included for maintaining the readership of the Journal.

 We included it as follows: “Moreover, considering the specific application, under the assumption of connecting the components in a common DC bus, a buck/boost converter with efficiency of 0.95 was taken into account for both storage units.”, in Section 4.1.

- Cost and Energy density of FESS shall be presented, as shown on pg. 4 for SCs.

 Ok, we added the costs related to FESS.

- The sizing of energy storage is not clear, this data shall be added that could show the purpose of the study is fully covered using FESS/HESS.

 Table 6 reassumes the final sizing of the HESS components, according to the methodology developed, as described in Section 5.

- Power converters control modelling and used topologies are also missing in the study. It would be beneficial for the readers to have this information available.

The aim of this preliminary study is to demonstrate the benefits introduced by HESS coupling to WECs. A schematic view of the electrical architecture is added in Figure 9a. Anyway, the design and control of the converters is out of the scope of the presented work and must be implemented in a future paper.

- Li-ion batteries are being complemented with Flywheel energy storage, this study lacks the information about the SOCs of the batteries and the power losses during the operation.

The HESS was modeled according to previous refs of the Authors (such as https://doi.org/10.3390/en11020396). Specifically, Flywheel is a low-speed mechanical flywheel, and it is implemented taking into account the aerodynamic and bearing losses in reference to the current rotational speed. The Li-ion battery is instead modeled by means of real measurements of open circuit voltages and internal resistances during charge/discharge at varying the state of charge. It is defined in Section 4.1 according to reviewers’ suggestions: “Specifically, the Li-ion battery and flywheel are implemented on the basis of their governing equations. Further details on the HESS implementation can be found in [1], [41-42]. Regarding the battery, open circuit voltage and internal resistance data were gathered by the authors, varying the state of charge in both charging and discharging phases, through a dedicated experimental campaign and implemented in the Simulink model. For what concerns the flywheel, the instantaneous delivered/absorbed power is computed taken into account the instantaneous aerodynamic and friction power losses according to the current flywheel speed value. “ As regards SOCs of the HESS, the Authors avoided to put many figures in the paper to improve the readability with a focus on the power smoothing aspects introduced by HESS since several simulations were carried out. Nevertheless, SOC trends can be found in the additional data at the link http://www.doi.org/10.5281/zenodo.5993310.

- Efficiency of the DC-DC power converter could be presented in buck and boost mode for the three sites investigated.

As previously described, the efficiency of the buck/boost converters is fixed at 0.95 for both the operating modes.

- Authors write “In this paper, HESS consisting of a Li-ion battery and a flywheel is coupled to a Wave Energy Converter (WEC) that operates in grid connected mode”. It is interesting what the model of flywheel-Li-ion battery HESS looks like. Constriction and connection principal to WEC.

According to reviewers’ suggestion, a schematic view of the electric connection to the WEC and the overall architecture is reported in Figure 9a.

-  Authors write on Page 3 “The Power Take-Off (PTO) subsystem, which converts the captured wave energy (by the prime mover) into electricity. The most common typologies are: hydraulic PTO, electro-mechanical PTO, linear generators, air turbine and low head water turbine”. Technical aspects: for example how to connect flywheels to linear generators? From my point of view, it is almost impossible to do. But even if it succeeds, it will be a complicated mechanical system that takes resources and becomes costly.

The flywheel is not directly connected to the linear generator. The HESS (i.e., consisting of FESS and BESS) is interfaced with the WECs by means of a common DC bus. Therefore, HESS operates absorbing/delivering power to the electric power produced by the WEC, without any interference with the PTO of the latter.

- It is good to adjust the scale in Fig.12 so that empty spaces are not so large.

We adjusted Figure 12 according to the suggestion.

- The authors have laid out in a very reasonable manner how simulations demonstrate that flywheel features can extend Li-ion battery lifespan, decreasing battery solicitations of more than 64% with respect to flywheel fluctuations.

Although this aspect is out of the paper’s scope, the Authors already demonstrated that HESS consisting by flywheel and Li-ion battery, thanks to the peak-shaving function operated by flywheel towards the battery, can strongly extend the battery lifespan, as found in our previous works: 1) https://doi.org/10.1016/j.energy.2019.02.143, 2) https://doi.org/10.3390/en12163138, 3) 10.1016/j.est.2020.102050.

- The article referenced some relevant references on HESS. However, few more references shall be included showing the HESS studies (Li-on Batteries/SCs/Flywheel/Fuel Cells).

We added refs. [31-35] as other noteworthy studies on hybrid energy storage systems.

- An overall overview of the Electrical power system used in this study shall be included.

As previously mentioned, a schematic overview of the electrical power system is reported in Figure 9a.

- The paper presents an interesting approach to reducing power fluctuations. It is a good platform for evaluating offshore experiments.
- Data is provided in Matlab. However, reproducing the experiment in more detail would significantly improve the paper. The paper is relevant for indexing.

We already provided and explained all the data for simulation reproduction, as annexed to the paper.

# Reviewer report: Reviewer response for version 1

**DOI:** 10.21956/openreseurope.15154.r28907

**Published:** 2022-04-12

**Authors:** Luigi Piegari, Marzio Barresi

**Affiliations:** 1 Polytechnic University of Milan, Milan, Italy

The paper deals with the use of hybrid energy storage systems with wave energy converters to improve the power quality of wave generators.

 Even if the idea is interesting, in the opinion of the reviewers, the paper does not completely achieve the claimed goals. It is worth noting that several data are provided in Matlab environment. Nevertheless, the minimum data to reproduce the experiment should be explained in the text, since using data already produced is very difficult and time-spending.

 The major points that should be addressed are detailed in the following:

- The efficiency of the storage units is given as a final data but it is not clear how it has been obtained. Indeed, there is no detailed model of the storages. The model used to simulate the storage units and their efficiencies should be provided.
- A cost analysis is necessary to understand the results. Indeed, for the data reported in the paper, FESS is much less efficient than batteries. Therefore, the additional cost related to energy losses should be accounted for to verify if this solution is better than using a bigger battery substituting it at the end of life.
- the size of the FESS is established only based on the power ramp. The battery power is obtained to complement the required power. There is no information about the energy sizing of each storage. This is very important to understand if the storages can accomplish their mission without being fully charged or discharged. The analysis of the state of charge during the simulated days should be added to the paper.
- Other sizing procedures should be studied and results compared with the proposed one. Indeed, the optimal size of flywheel and batteries could be different from the one obtained with the simple procedure proposed in the paper (i.e. FESS power = required power ramp).
- BESS and FESS need a power converter to connect to the grid. How the efficiency of these converters has been taken into account?
- An overall scheme of the electrical power system plant should be added to the paper.

Some other minor comments are the following:

- FESS can experience problems if subjected to high accelerations. Usually, a platform in the sea can be subjected to these accelerations. Some considerations about the feasibility of using FESS for this application could be interesting.
- Some details about the implementation of the control could improve the readability. For example, are the authors supposing that the power converter can precisely follow the references? How this hypothesis can affect the final results?
- When acronyms are defined uppercase should be avoided (e.g. “ *Wave Energy Converters (WECs)*” should be “ *wave energy converters (WECs)*”).
- In the sections, before starting with subsections, a few sentences to introduce the topic could be added.
- At pag. 4, col. 1, cost and energy density of SCs are compared with those of FESS. For a fair comparison, also the cost and the energy density of the FESS should appear in the text.
- Fig. 8 is difficult to read and its content is not very significant. Probably it could be eliminated leaving space for figures reporting more interesting data (e.g. the SOCs of the HESS during operation, the losses of the storages…).
- In Table 5, “A” (uppercase) is reported twice (with two different values). Likely, one of the two should be “a” (lowercase).

Is the study design appropriate and does the work have academic merit?

Partly

Is the work clearly and accurately presented and does it cite the current literature?

Yes

If applicable, is the statistical analysis and its interpretation appropriate?

I cannot comment. A qualified statistician is required.

Are all the source data underlying the results available to ensure full reproducibility?

Partly

Are the conclusions drawn adequately supported by the results?

Partly

Are sufficient details of methods and analysis provided to allow replication by others?

No

Reviewer Expertise:

Power electronics for renewables, batteries and automotive, electrical drives, electrochemical storage modeling

We confirm that we have read this submission and believe that we have an appropriate level of expertise to confirm that it is of an acceptable scientific standard, however we have significant reservations, as outlined above.

# Response

**Published:** 2022-04-28

**Authors:** Federico Gallorini

**Affiliations:** VGA srl, Italy

POINT TO POINT ANSWERS TO REVIEWER (Reviewer comments in italics)

*The major points that should be addressed are detailed in the following:*

- *The efficiency of the storage units is given as a final data but it is not clear how it has been obtained. Indeed, there is no detailed model of the storages. The model used to simulate the storage units and their efficiencies should be provided.*

As written in the paper, the described dynamic model was developed and detailed in our previous papers. Specifically, the li-ion battery and flywheel were implemented on the basis of mathematical equations. For what concerns flywheel, the instantaneous delivered or absorbed power was computed taken into account the instantaneous aerodynamic and friction power losses according to the current flywheel speed value. Moreover, considering the specific application, under the assumption of connecting the components in a common DC bus, a buck/boost converter efficiency of 0.95 was taken into account both for battery and flywheel. For sake of clarity, we can add the following part to the “Modeling considerations” section:

**“Specifically, the li-ion battery and flywheel are implemented on the basis of their governing equations. Further details on the HESS implementation can be found in [1], [41-42]. For what concerns the flywheel, the instantaneous delivered/absorbed power is computed taken into account the instantaneous aerodynamic and friction power losses according to the current flywheel speed value. Moreover, considering the specific application, under the assumption of connecting the components in a common DC bus, a buck/boost converter efficiency of 0.95 was taken into account for both the storage units.”  ** 

- *A cost analysis is necessary to understand the results. Indeed, for the data reported in the paper, FESS is much less efficient than batteries. Therefore, the additional cost related to energy losses should be accounted for to verify if this solution is better than using a bigger battery substituting it at the end of life.*

Although we understand the importance of a cost analysis of the hybrid energy storage integration in wave energy converters for power smoothing, the paper’s main aim is not addressed to such evaluation. Anyway, we already assessed the benefits of flywheel-li-ion battery integration into a renewable power plant in our previous several papers (INS. RIF.). Specifically, flywheel’s peak shaving function acting on the battery allows to reduce harmful power spikes strongly enhancing battery lifespan. We proved such results both by applying rainflow cycle counting algorithm ( https://www.mdpi.com/1996-1073/12/16/3138; https://doi.org/10.1016/j.est.2020.102050) and conducting experimental tests ( https://doi.org/10.1016/j.energy.2019.02.143).

- *The size of the FESS is established only based on the power ramp. The battery power is obtained to complement the required power. There is no information about the energy sizing of each storage. This is very important to understand if the storages can accomplish their mission without being fully charged or discharged. The analysis of the state of charge during the simulated days should be added to the paper.*

The same procedure to determine power and capacity of the HESS components was followed, as reported in the paper, Section 5: **“a first assumption in reference to both storage devices is made starting from the cumulative distribution of the power ramp calculated according to eq. (3). It is noted that the storage devices are customized one at a time, aiming to avoid/minimize saturation in power and capacity; so, the flywheel rating (both in power and capacity) is adjusted first, assuming a very large battery, since the flywheel is more adequate to fast response operation (i.e. power smoothing). The capacity and maximum power of the flywheel are determined so that the saturation objective is achieved. Further, the capacity of the battery is adjusted (so, considering the C-rate also the power), following the same objective in terms of saturation.”** Therefore, storage units accomplish their mission without being fully charged or discharged during the daily simulations.

- *Other sizing procedures should be studied and results compared with the proposed one. Indeed, the optimal size of flywheel and batteries could be different from the one obtained with the simple procedure proposed in the paper (i.e. FESS power = required power ramp).*

The presented procedure for sizing was developed for this specific application in order to properly work with the implemented power management algorithm (SPSA). In fact, the power management strategy pursues its objective of reducing power oscillations produced by the WECs and providing a smoothed instantaneous power profile at the point of common coupling. Such an algorithm acts on the instantaneous power ramp of the generation, mitigating the oscillations by means of requiring/providing a certain amount of power to the storage units. Therefore, comparing the sizing procedure of this work with other ones is outside of the paper’s purpose.

- *BESS and FESS need a power converter to connect to the grid. How the efficiency of these converters has been taken into account?*

How it is pointed out in the first answer, we assumed an efficiency equal to 0.95 for the HESS converters and it is implemented in the dynamic model acting on the instantaneous power delivered/provided by the storage components, increasing the amount of power sent to the flywheel and battery during their charge and decreasing the power provided by the storage units during their discharge.

- An overall scheme of the electrical power system plant should be added to the paper.

As reviewers suggest, we can provide the electrical architecture of the plant in the paper for sake of clarity (see the following figure). This figure can be added in Figure 9.

*Some other minor comments are the following:*

- *FESS can experience problems if subjected to high accelerations. Usually, a platform in the sea can be subjected to these accelerations. Some considerations about the feasibility of using FESS for this application could be interesting.*

We thank the reviewers for pointing out this issue. Nevertheless, to avoid such problems and, at the same time, to better interface the system with the main grid, the installation of the storage units was assumed to be on the ground.

- *Some details about the implementation of the control could improve the readability. For example, are the authors supposing that the power converter can precisely follow the references? How this hypothesis can affect the final results?*

Since the model simulates a complex integrated system, we did not enter in details concerning the power converters, only considering their efficiencies. Thus, the dynamic model of the power converters was not included.

- *When acronyms are defined uppercase should be avoided (e.g. “Wave Energy Converters (WECs)” should be “wave energy converters (WECs)”).*

 Ok, we can modify the acronyms according to the reviewers’ suggestions if the editor agrees.

- *In the sections, before starting with subsections, a few sentences to introduce the topic could be added.*

As reviewers suggested, we can introduce in the revised version of the paper the topics with a few sentences for the missing sections of the paper.

- *At pag. 4, col. 1, cost and energy density of SCs are compared with those of FESS. For a fair comparison, also the cost and the energy density of the FESS should appear in the text.*

We can add in the revised paper the values both for flywheel and supercapacitors. As indicated in [21], the total project cost is 75,000 $/kWh for SCs and 11,520 $/kWh for FESS.

  

- *12. Fig. 8 is difficult to read and its content is not very significant. Probably it could be eliminated leaving space for figures reporting more interesting data (e.g. the SOCs of the HESS during operation, the losses of the storages…).*

We think that the figure illustrates the intrinsic characteristics of the wave energy sector and the variability with respect to the sites.

- *13. In Table 5, “A” (uppercase) is reported twice (with two different values). Likely, one of the two should be “a” (lowercase).*

The second row (a) and third row (c) of Table 5 are to be in lowercase.

# Response

**Published:** 2023-08-01

**Authors:** Linda Barelli

**Affiliations:** Department of Engineering, Universita degli Studi di Perugia, Perugia, Umbria, Italy

POINT TO POINT ANSWERS TO REVIEWERS The paper deals with the use of hybrid energy storage systems with wave energy converters to improve the power quality of wave generators. Even if the idea is interesting, in the opinion of the reviewers, the paper does not completely achieve the claimed goals. It is worth noting that several data are provided in Matlab environment.  The major points that should be addressed are detailed in the following:

1. The efficiency of the storage units is given as a final data but it is not clear how it has been obtained. Indeed, there is no detailed model of the storages. The model used to simulate the storage units and their efficiencies should be provided.

As written in the paper, the described dynamic model was developed and detailed in our previous papers. Specifically, the li-ion battery and flywheel were implemented on the basis of mathematical equations. For what concerns flywheel, the instantaneous delivered or absorbed power was computed taken into account the instantaneous aerodynamic and friction power losses according to the current flywheel speed value. Moreover, considering the specific application, under the assumption of connecting the components in a common DC bus, a buck/boost converter efficiency of 0.95 was taken into account both for battery and flywheel. For sake of clarity, we added the following part to the “Modeling considerations” section: “ *Specifically, the li-ion battery and flywheel are implemented on the basis of their governing equations. Further details on the HESS implementation can be found in* *[1], [41-42]* *. For what concerns the flywheel, the instantaneous delivered/absorbed power is computed taken into account the instantaneous aerodynamic and friction power losses according to the current flywheel speed value. Moreover, considering the specific application, under the assumption of connecting the components in a common DC bus, a buck/boost converter efficiency of 0.95 was taken into account for both the storage units.”*   

1. A cost analysis is necessary to understand the results. Indeed, for the data reported in the paper, FESS is much less efficient than batteries. Therefore, the additional cost related to energy losses should be accounted for to verify if this solution is better than using a bigger battery substituting it at the end of life.

Although we understand the importance of a cost analysis of the hybrid energy storage integration in wave energy converters for power smoothing, paper’s main aim is not addressed to such evaluation. Anyway, we already assessed the benefits of flywheel-li-ion battery integration into a renewable power plant in our previous several papers (INS. RIF.). Specifically, flywheel’s peak shaving function acting on the battery allows to reduce harmful power spikes strongly enhancing battery lifespan. We proved such results both applying rainflow cycle counting algorithm ( https://www.mdpi.com/1996-1073/12/16/3138; https://doi.org/10.1016/j.est.2020.102050) and conducing experimental tests ( https://doi.org/10.1016/j.energy.2019.02.143).

1. the size of the FESS is established only based on the power ramp. The battery power is obtained to complement the required power. There is no information about the energy sizing of each storage. This is very important to understand if the storages can accomplish their mission without being fully charged or discharged. The analysis of the state of charge during the simulated days should be added to the paper.

The same procedure to determine power and capacity of the HESS components was followed, as reported in the paper, Section 5: “ *a first assumption in reference to both storage devices is made starting from the cumulative distribution of the power ramp calculated according to eq. (3). It is noted that the storage devices are customized one at a time, aiming to avoid/minimize saturation in power and capacity; so, the flywheel rating (both in power and capacity) is adjusted first, assuming a very large battery, since the flywheel is more adequate to fast response operation (i.e. power smoothing). The capacity and maximum power of the flywheel are determined so that the saturation objective is achieved. Further, the capacity of the battery is adjusted (so, considering the C-rate also the power), following the same objective in terms of saturation*.” Therefore, storage units accomplish their mission without being fully charged or discharged during the daily simulations.

1. Other sizing procedures should be studied and results compared with the proposed one. Indeed, the optimal size of flywheel and batteries could be different from the one obtained with the simple procedure proposed in the paper (i.e. FESS power = required power ramp).

The presented procedure for sizing was developed for this specific application in order to properly work with the implemented power management algorithm (SPSA). In fact, the power management strategy pursues its objective of reducing power oscillations produced by the WECs and providing a smoothed instantaneous power profile at the point of common coupling. Such algorithm acts on the instantaneous power ramp of the generation, mitigating the oscillations by means of requiring/providing a certain amount of power to the storage units. Therefore, comparing the sizing procedure of this work with other ones, it is outside of paper’s purpose.

1. BESS and FESS need a power converter to connect to the grid. How the efficiency of these converters has been taken into account?

 How it is pointed out in the first answer, we assumed an efficiency equal to 0.95 for the HESS converters and it is implemented in the dynamic model acting on the instantaneous power delivered/provided by the storage components, increasing the amount of power send to the flywheel and battery during their charge and decreasing the power provided by the storage units during their discharge.

1. An overall scheme of the electrical power system plant should be added to the paper.

As reviewers suggest, we provided the electrical architecture of the plant in the paper for sake of clarity (see as Figure 9a).   Some other minor comments are the following:

1. FESS can experience problems if subjected to high accelerations. Usually, a platform in the sea can be subjected to these accelerations. Some considerations about the feasibility of using FESS for this application could be interesting.

We thanks the reviewers for pointing out this issue. Nevertheless, to avoid such problems and, at the same time, to better interface the system with the main grid, the installation of the storage units was assumed to be on the ground.

1. Some details about the implementation of the control could improve the readability. For example, are the authors supposing that the power converter can precisely follow the references? How this hypothesis can affect the final results?

Since the model simulates a complex integrated system, we did not enter in details concerning the power converters, only considering their efficiencies. Thus, the dynamic model of the power converters was not included.

1. When acronyms are defined uppercase should be avoided (e.g. “Wave Energy Converters (WECs)” should be “wave energy converters (WECs)”).

 Ok, we modified the acronyms according to reviewers’ suggestion.

1. In the sections, before starting with subsections, a few sentences to introduce the topic could be added.

 As reviewers suggested, we introduced the topics with a few sentences for the missing sections of the paper.

1. At pag. 4, col. 1, cost and energy density of SCs are compared with those of FESS. For a fair comparison, also the cost and the energy density of the FESS should appear in the text.

We added in the paper the total project costs both for flywheel and supercapacitors, as indicated in [21].

1. In Table 5, “A” (uppercase) is reported twice (with two different values). Likely, one of the two should be “a” (lowercase).

We found a mistake in the table. We modified the second (a) and third (c) term of Table 5 with lowercase.
